# Effects of acute, subacute, and chronic exercise on plasma s-Klotho levels: a systematic review and meta-analysis

**DOI:** 10.1007/s13105-026-01182-2

**Published:** 2026-05-02

**Authors:** Túlio Medina Dutra de Oliveira, Diogo Carvalho Felício, Taynara da Silva Ribeiro, José Elias Filho, Francisco José Amaro-Gahete, Giovani Bernardo Costa, Jorge Willian Leandro Nascimento, Maycon de Moura Reboredo, Carla Malaguti

**Affiliations:** 1 Postgraduate Program in Health, Faculty of Medicine, Federal University of Juiz de Fora, Juiz de Fora, 36038-330 Brazil; 2 Postgraduate Program in Rehabilitation Sciences and Physical-Functional Performance, Faculty of Physiotherapy, Federal University of Juiz de Fora, Juiz de Fora, 36038-330 Brazil; 3 Department of Medical Physiology, Faculty of Medicine, University of Granada, 18010 Granada, Spain; 4 University Hospital, Federal University of Juiz de Fora, Juiz de Fora, 36038-330 Brazil; 5 Department of Pharmacology, Institute of Biological Sciences, Federal University of Juiz de Fora, Juiz de Fora, 36036-330 Brazil; 6 Universidade Federal de Juiz de Fora, Campus UFJF, São Pedro, Juiz de Fora, MG 36036-900 Brazil

**Keywords:** Klotho, Exerkine, Exercise, Physical training, Chronic disease

## Abstract

Exercise exerts beneficial effects on multiple physiological systems, influencing biomarkers associated with aging processes and chronic diseases. This systematic review and meta-analysis aimed to evaluate the acute, subacute, and chronic effects of exercise on s-Klotho levels in healthy individuals and patients with chronic diseases. A comprehensive search was conducted in electronic databases. Included studies were randomized and non-randomized studies assessing the effects of aerobic, resistance, or combined exercise on s-Klotho levels. Interventions were classified as acute (single session), subacute (< 12 weeks), or chronic (≥ 12 weeks). Risk of bias was assessed using RoB 2 and ROBINS-I tools, and quality of evidence was evaluated with the GRADE framework. Forty-one studies were included in the qualitative synthesis, and 30 in the meta-analysis, comprising 2,765 participants (18–85 years). Twenty-five involved healthy individuals, while 21 included patients with chronic diseases. Acute and subacute aerobic exercise increased s-Klotho levels in healthy individuals (SMD 0.69; 95%CI 0.41–0.97) and diseased populations (SMD 0.62; 95%CI 0.11–1.12). Chronic exercise (12–36 weeks) showed significant increases in healthy (SMD 0.57; 95%CI 0.33–0.82) and diseased populations (SMD 1.51; 95%CI 0.87–2.16). Resistance exercise thrice weekly demonstrated the highest effect (SMD 1.60; 95%CI 0.81–2.38). Most studies had high risk of bias, with quality of evidence ranging from very low to moderate. Exercise significantly increases s-Klotho levels, with acute and subacute aerobic sessions benefiting healthy and diseased populations, while chronic resistance exercise elicited greater responses in diseased populations. Despite promising findings, low methodological quality suggests cautious interpretation.

**Supplementary Information:**

The online version contains supplementary material available at 10.1007/s13105-026-01182-2.

## Key points

Exercise significantly increases circulating s-Klotho in healthy and clinical populations.

Chronic exercise (12–36 weeks) enhances s-Klotho more than acute or subacute protocols.

Resistance exercise three times per week induces the largest increase in s-Klotho levels.

**Supplementary Information:**

The online version contains supplementary material available at 10.1007/s13105-026-01182-2.

## Introduction

The klotho gene, which encodes the anti-aging protein called Klotho, was discovered in 1997 by Kuro-o et al. through an incidental silencing of the gene in mice [41]. This genetic alternation led to the development of multisystem organ failure and a significantly reduced lifespan, resembling the characteristics of premature aging in humans [41]. Klotho possesses a transmembrane-bound co-receptor form for fibroblast growth factor (FGF23) [88] or a soluble form (s-Klotho) derived from proteolytic cleavage of transmembrane form [15].

s-Klotho is present in blood, urine and cerebrospinal fluid [40, 77] and exerts multiple physiological functions, such as reducing oxidative stress in cells, suppressing chronic inflammation, or regulating mineral homeostasis [30, 39]. s-Klotho can act as a paracrine or endocrine mediator through the modulation of growth factors and cytokines, including insulin, insulin-like growth factor-I, transforming growth factor-β, Wnt signaling and interferon-gamma, which are associated with cellular senescence and the aging process in mice [42, 47, 86].

Recent literature has demonstrated a significant association between s-Klotho levels and several health outcomes across representative populations. Indeed, reduced s-Klotho levels correlate with increased risk of all-cause mortality, cardiovascular disease, diabetes, osteoporosis, low cognitive performance, frailty and low physical function among both healthy adults and the elderly [7, 34, 37, 44, 45, 75, 91].

The modulation of klotho protein synthesis through skeletal muscle contraction represents an intriguing mechanism that may shed light on the anti-aging effects of exercise [5]. However, it remains unclear whether skeletal muscle contraction directly induces the synthesis of s-Klotho or if this process is mediated by known myokines secreted by skeletal muscle (e.g., irisin or interleukin-6) which may promote klotho synthesis in organs such as the kidneys or brain [8]. Although klotho is not yet formally classified as a myokine, emerging evidence suggests that it may meet the criteria for such a designation [2–4]. In this regard, understanding the role of skeletal muscle activity and exercise as potential regulators of klotho expression is important for the development of targeted, specific rehabilitation programs designed to counteract the effect of aging on human´s health.

Therefore, this systematic review aims to assess and describe the effect of acute, subacute and, chronic impact of exercise on s-Klotho levels in both healthy individuals and patients with chronic diseases.

## Methods

The protocol was prospectively registered in the International Prospective Register of Systematic Reviews (PROSPERO) (Registration number: CRD42020208002, December 2023). This systematic review was conducted in accordance with the recommendations of the Cochrane Handbook [10] and reported following the Preferred Reporting Items for Systematic Reviews and Meta-Analyses (PRISMA) guidelines [63].

### Search strategy

The search was carried out in January 2024 and updated in March 2025 using the following databases: PubMed/MEDLINE, Embase, CINAHL, CENTRAL, Scopus, Web of Science, LILACS and SciELO. We strictly followed the recommendations of each database and used descriptors and their variations, combining them with the Boolean operator "AND". There were no restrictions related to publication date or language. The selected articles were manually screened to identify additional studies from the reference lists. The complete search strategy is presented in the Supplementary Material 1.

### Eligibility criteria

Clinical studies involving human participants were included. Randomized controlled trials (RCT) and non-randomized studies (NRS) investigating the impact of exercise (e.g., aerobic, resistance, neuromuscular electrical stimulation, Pilates, or maximal exercise testing) on s-Klotho levels, were included. Exercise interventions were classified according to duration. Acute exercise was considered as a short or singular period of exercise while chronic exercise was understood as interventions performed over a period of 12 weeks or more [66]. Subacute exercise was categorized as those falling between the acute and chronic periods. No restrictions were applied regarding population age or health status, as exercise can modulate klotho expression across diverse populations [3, 14, 18, 23, 25]. Studies that compared different modalities of (i) exercise versus usual care, (ii) exercise versus non-intervention or (iii) pre- and post-intervention comparisons (single-arm) were included.

Studies combining exercise interventions with the use of medication or hormonal therapy were excluded. Moreover, studies were also excluded if they (i) failed to report direct data on s-Klotho levels, (ii) focused solely on aspects related to the klotho gene or the β-klotho receptor, (iii) included participants with severe acute conditions such as acute myocardial infarction, decompensated heart failure, recent stroke, severe pneumonia or respiratory failure and severe systemic infections and (iv) involved pre-clinical or animal models.

### Selection of studies and data extraction

Two reviewers (TMDO and GBC) exported the studies identified using the aforementioned search strategy to EndNote X9 (Thomson Reuters, Philadelphia, USA) for the removal of duplicates. Subsequently, two reviewers (TMDO and GBC) independently screened titles and abstracts for potential eligibility. Full-text articles were assessed to establish final inclusion in the review. Any disagreements between reviewers were resolved through discussion or by arbitration with a third reviewer (CM). Data extraction, including names of the authors, year of publication, study design, age, population, sample size, exercise protocol, duration and measurement point, was also performed by two reviewers (TMDO and TSR). Disagreements between reviewers were resolved through discussion, or arbitration by a third reviewer (CM).

### Risk of bias assessment

The Cochrane Risk of Bias 2 (RoB 2) and the Risk of Bias in Non-randomized Studies—of Interventions (ROBINS-I) tools were used to evaluate the methodological quality of the included RCTs and NRSs, respectively. The RoB 2 tool assesses methodological quality across five domains: the randomization process, deviations from intended interventions, missing outcome data, outcome measurements, and reporting. Studies were classified as having (i) a low risk of bias if all domains were judged as low risk, (ii) a moderate risk of bias if one or more domains were judged as presenting some concerns, and (iii) a high risk of bias if one or more domains were judged as high risk of bias [83]. The ROBINS-I tool evaluates methodological quality across seven domains: confounding, selection of participants, classification of interventions, deviations from intended interventions, missing data, measurement of outcomes, and selection of the reported result. The risk of bias was classified into five categories: (i) low risk, (ii) moderate risk, (iii) serious risk (iv) critical risk of bias and (v) no information [82].

The assessment of each domain, for both RoB 2 and ROBINS-I, was based on consensus reached through the independent evaluation of two reviewers (TMDO and TRS). In the event of disagreement, arbitration was provided by a third reviewer (CM). Publication bias was assessed using a funnel plot.

### Quality of evidence

The Grading of Recommendations, Assessment, Development and Evaluation (GRADE) system [27] was used to assess the overall quality of the evidence. GRADE guidelines evaluate five domains: risk of bias, inconsistency, indirectness, imprecision, and publication bias. The quality of the evidence was downgraded according to the criteria of study design, risk of bias, inconsistency, indirectness, imprecision and publication bias. For each domain, the reasons for downgrading were as follows: (1) study design: one level of NRS; (2) risk of bias: one level if the overall assessment presented some concerns; two levels if the overall assessment was high risk of bias; (3) inconsistency: one level if there was minimal overlap in the effect estimates among the studies; two levels if the confidence intervals of the effect estimates did not overlap; (4) indirectness: one level if there was indirectness from one source (population, intervention, comparison, outcome); two levels if there was indirectness from more than one source; (5) imprecision: one level if the total sample size (sum of both groups) was less than 400; two levels when the CI around the effect estimate included meaningful effect and no effect; (6) publication bias: one level if publication bias was suspected. The quality of evidence is classified into four levels: very low, low, moderate, and high. The assessment was performed using the GRADEpro software (https://gradepro.org/).

### Statistical analysis

A random-effects meta-analysis was performed due to expected heterogeneity between studies related to health status and modalities of exercise. Continuous data were expressed as standardized mean differences (SMDs) with 95% confidence intervals (CI95%). Effect sizes were classified as minimal, small, medium and large for SMD values of < 0.2, 0.2 to 0.5, 0.5 to 0.8, and > 0.8, respectively [12]. Meta-analysis were conducted using pre- and post-intervention data for NRSs and post-intervention data from the intervention and control groups for RCTs. Studies with multiple treatment groups were analyzed as independent studies. In cases where the control group sample was repeated within the same forest plot, the sample size was halved. Heterogeneity was assessed through visual inspection of the forest plots and quantified using the I^2^ statistic [10]. I^2^ values were interpreted as follows: 0–40% suggests that heterogeneity might not be important; 30–60% represents moderate heterogeneity; and 50–90% indicates substantial heterogeneity, with *p < *0.10. Subgroup analysis was performed based on variables that could influence the s-Klotho response to exercise and could contribute to heterogeneity: health status (with and without disease); exercise modality (aerobic, resistance, or concurrent); duration of the exercise protocol (acute, subacute or chronic); and weekly frequency. For the subgroup of those subjects with diseases, if more than one study assessed the same health condition, the data were pooled. To assess the impact of each study on the pooled results, a leave-one-out sensitivity analysis was conducted. The risk of publication bias was evaluated through inspection of the funnel plot and employing the Egger test when 10 or more studies reported the same outcome. Statistical significance was defined as *p < *0.05. All analyses were conducted using RevMan 5.4 software.

## Results

### Study selection

**Fig. 1:**
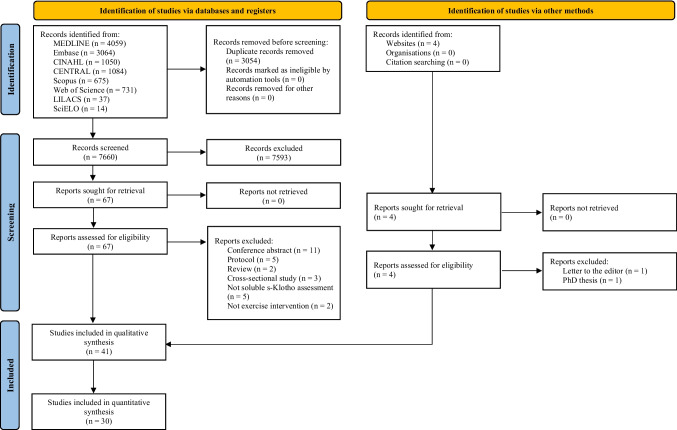
PRISMA flow diagram which included searches of databases, registers and other sources

The literature search identified 10,714 articles. The PRISMA flow diagram summarizes the results of the literature search (Fig. 1). Forty-one studies were included in the qualitative analysis and 30 provided enough information to warrant inclusion in the meta-analysis.

### Population characteristics

Twenty-five studies included healthy subjects [1, 3, 6, 18, 25, 32, 35, 53, 54, 56–60, 66, 68, 71, 73, 74, 80, 81, 84, 85, 89, 92] and 21 included subjects with some form of disease [9, 13, 14, 16–19, 23–25, 35, 43, 49, 55, 61, 64, 67, 72, 74, 78, 80]. The investigated pathological conditions were chronic kidney disease [14, 16, 17, 19, 61], coronary artery disease [49, 72, 74], type II diabetes mellitus [24, 25, 35, 67], obesity or being overweight [13, 78, 80], chronic obstructive pulmonary disease [9, 64], acute coronary syndrome [43], rheumatoid arthritis [18], systemic arterial hypertension [35], asthma [55], and adults at risk for Alzheimer's disease [23]. There were 2,765 subjects with ages ranging from 18 to 85 years. The included studies were published between 2014 and 2024, reflecting the years of publication of the eligible studies identified through the systematic search, since no restriction on publication year was applied.

### Exercise characteristics

Regarding the exercise modality, 29 studies prescribed aerobic [1, 3, 13, 18, 23–25, 32, 35, 43, 53–55, 57–60, 64, 66–68, 71–74, 84, 85, 89, 92], 13 resistance [9, 14, 16, 17, 24, 32, 49, 56, 57, 61, 78, 81, 92] and 6 concurrent exercise [3, 6, 19, 60, 80, 89]. Details of the protocols are presented in Table 1.

**Table 1:** Characteristics of the included studies

Study	Design	Age (years)	Population and sample size	Exercise protocol		Duration/measure point
				Modality	Intensity	
Acute studies						
Aczel 2023	NRS	37 to 85	Trained and sedentary adults and older people *n* = 202	Aerobic exercise test	80% of the estimated maximum heart rate	1 day/24–48 h after exercise session
Dundar 2022	NRS	19.8 ± 2.5	Trained athletes *n* = 20	Aerobic exercise test	Submaximal; 200 W to 250W	1 day/two and 48 h after the exercise test
Ercan 2023	NRS	44.7 ± 8	Rheumatoid arthritis and healthy subjects *n* = 80	Aerobic exercise test	60–80% of maximal HR	1 day/immediately after 30 min of exercise
Goulet 2024	NRS	61.3 ± 6.1	Type II diabetes and healthy subjects *n* = 30	Aerobic exercise test	Low, moderate, and high-intensity at fixed rates of metabolic heat production (150, 200, and 250 W/m^2^)	1 day/immediately after 30 min of exercise
Iturriaga 2021	NRS	29.9 ± 9	Healthy and physical active men *n* = 82	Group 1: aerobic exercise test Group 2: resistance exercise trial	Aerobic exercise test: 75% of VO_2_max Resistance exercise: > 70% of RPE	1 day/immedialety, 24, 48, and 72 h after 50 min of exercise
King 2023	NRS	59.6 ± 4.1	Older with and without hypertension and type II diabetes *n* = 31	Aerobic exercise test	4.8 km/h, 2.5% incline, fixed metabolic rate of ~ 3.4 METs	1 day/immedialety and 60 min after 180 min of exercise test
Morishima 2021	NRS	20.4 ± 0.3	Untrained men *n* = 12	Resistance exercise trial	~ 80% of predicted 1RM	1 day/immediately, 30 and 60 min after an exercise session
Morishima 2022	NRS	20.4 ± 0.3	Healthy men *n* = 12	Group 1: aerobic exercise test Group 2: resistance exercise trial	Aerobic exercise test: RPE 11–13 on Borg scale Resistance exercise trial: 70% 1-RM	1 day/immediately, 30 and 60 min after an exercise session
Mostafidi 2016	NRS	18 to 27	Trained athletes *n* = 30	Aerobic exercise session	Not reported	1 day/12 h after a sports exercise session
Nava 2018	NRS	29 ± 7	Physically-active subjects *n* = 10	Aerobic exercise test	Maximal HR ± 10 bpm of age-predicted; VO_2_ plateau of ≤ 150 mL/min; RPE > 17	1 day/48 h after a maximum exercise capacity test
Rahimi 2018	NRS	31.5 ± 7.8	Healthy non-athlete women *n* = 10	Aerobic exercise test	High intensity; the speed increases by 0.8 km/h and the incline by 2% each stage	1 day/24 h after exercise
Saghiv 2015	NRS	23 to 60	Healthy adults and older *n* = 200	Aerobic exercise test	Increase intensity until reaching age-predicted maximal HR	1 day/after exercise
Tan 2018	NRS	45 to 51	Healthy adults *n* = 10	Aerobic exercise test	85% of estimated maximum HR	1 day/immediately, 30 min, 240 min and 1 week after the last exercise test
Subacute studies						
Ghadamyari 2024	RCT	56.6 ± 1.3	Type II diabetes women *n* = 30	Group 1: aerobic endurance exercise Group 2: resistance exercise	Aerobic endurance exercise: gradually increased from moderate to high intensity (50–75% HRmaxR) Resistance exercise: progressed from moderate to high intensity (50–75% 1-RM)	8 weeks/48 h after the last exercise session
Lázaro 2024	NRS	56 (47–63)	Acute coronary syndrome *n* = 174	Aerobic exercise	Not reported	8 weeks/8th week
Middelbeek 2021	RCT	48 ± 5	Healthy sedentary men *n* = 22	Group 1: aerobic sprint interval exercise Group 2: aerobic continous exercise	Aerobic sprint interval exercise: high intensity; 6 × 30 s all-out sprints Aerobic continous exercise: moderate intensity; 60% VO_2peak_	2 weeks/48 h after the last exercise session
Pako 2017	NRS	63.3 ± 8.8	Chronic obstructive pulmonary disease *n* = 19	Aerobic exercise	Not reported	3 weeks/3rd week
Rajabi 2018	RCT	51.5 ± 5.4	Type II diabetes postmenopausal women *n* = 24	Aerobic exercise	45% to 70% of HR reserve	8 weeks/8th week
Rangraz 2023	NRS	22 to 30	Sedentary women *n* = 20	Aerobic exercise	55% to 70% of maximal HR	6 weeks/6th week
Saghiv 2019	RCT	27 ± 1.1	Adult males *n* = 60	Group 1: low intensity aerobic exercise Group 2: high intensity aerobic exercise	Low intensity aerobic exercise: below anaerobic threshold at 40–50% VO_2_max High intensity aerobic exercise: above anaerobic threshold at 65–70% VO_2_max	8 weeks/8th week
Chronic studies						
Amaro-Gahete 2024	RCT	22.2 ± 2.2	Young adults *n* = 144	Group 1: moderate aerobic and resistance exercise Group 2: vigorous aerobic and resistance exercise	Moderate aerobic and resistance exercise: aerobic exercise at 60% of reserve HR and resistance exercise at 50% of 1-RM Vigorous aerobic and resistance exercise: aerobic exercise at 60–80% of reserve HR and resistance exercise at 70% of 1-RM	24 weeks/24th week
Amaro-Gahete 2019	RCT	53.4 ± 5.0	Sedentary middle-aged adults *n* = 74	Group 1: aerobic and resistance exercise Group 2: short and long high intensity aerobic interval exercise Group 3: short and long aerobic interval exercise + WB-EMS	Aerobic and resistance exercise: 60% to 65% of reserve HR and 40% to 50% 1-RM Short and long aerobic interval exercise: high intensity; > 95% VO_2_ max and > 120% VO_2_ max Short and long aerobic interval exercise + WB-EMS: high intensity; 80 mA to 100 mA; duty cycle 50–99%	12 weeks/12th week
Boeselt 2017	NRS	65.3 ± 8.3	Chronic obstructive pulmonary disease *n* = 37	Resistance exercise	35–75% of maximal muscle strength	12 weeks/12th and 24th weeks
Castro 2024	RCT	57.5 ± 4.0	Chronic kidney disease (end-stage) *n* = 78	Group 1: resistance exercise Group 2: cluster-set resistance exercise	Resistance exercise: RPE 5–6 progressing to RPE 7–8 Cluster-set resistance exercise: RPE 5–6 progressing to RPE 7–8	24 weeks/24 h after the last exercise session
Collins 2023	RCT	45.0 ± 7.9	Adults with overweight or obesity *n* = 152	Aerobic exercise	Moderate to vigorous; 250 min/week	18 and 36 weeks/18th and 36th weeks
Correa 2021	RCT	58.0 ± 6	Chronic kidney disease (stage 2) *n* = 105	Group 1: resistance exercise Group 2: resistance exercise + blood flow restriction	Resistance exercise: 50% to 70% 1-RM Resistance exercise + blood flow restriction: 30% to 50% 1-RM	18 weeks/18th week
de Araujo 2023	RCT	58 ± 4	Chronic kidney disease (stage 2) *n* = 31	Resistance exercise	3 to 8 on the OMNI scale	22 weeks/22th week
Fakhrpour 2020	RCT	61 ± 9	Chronic kidney disease (end-stage) *n* = 45	Aerobic and resistance exercise	Aerobic exercise: 12 to 14 on the modified Borg scale Resistance exercise: 40% to 65% 1-RM; 9 to 15 on the modified Borg scale	16 weeks/16th week
Gaitan 2021	RCT	65 ± 4.5	Middle-age adults at risk for Alzheimer’s disease *n* = 23	Aerobic exercise	Moderate to vigorous	26 weeks/26th week
Re 2023	RCT	62.6 ± 10.7	Coronary artery disease *n* = 55	Neuromuscular electrical stimulation	Low intensity electrical stimulation (50 Hz; 200 μs; 12-s on/6-s off)	12 weeks/12th week
Matsubara 2014	NRS	60 ± 1	Post menopausal women *n* = 69	Aerobic exercise	70% to 80% of maximal HR	12 weeks/12th week
Moraes-Ferreira 2022	NRS	36.1 ± 15.6	Asthma *n* = 21	Aerobic exercise	70% to 80% reserve heart rate and 6–7 on the modified Borg scale	9 weeks/9th week
Navarro-Lomas 2024	RCT	53.6 ± 4.9	Sedentary middle-aged adults *n* = 66	Group 1: aerobic and resistance exercise Group 2: short and long high intensity aerobic interval exercise Group 3: short and long aerobic interval exercise + WB-EMS	Aerobic and resistance exercise: 60% to 65% of reserve HR and 40% to 50% 1-RM Short and long aerobic interval exercise: high intensity; > 95% VO_2_ max and > 120% VO_2_ max Short and long aerobic interval exercise + WB-EMS: high intensity; 80 mA to 100 mA; duty cycle 50–99%	12 weeks/12th week
Neves 2021	RCT	56.3 ± 15.9	Chronic kidney disease (end-stage) *n* = 193	Group 1: dynamic resistance exercise Group 2: isometric resistance exercise	Dynamic resistance exercise: 6 to 8 on the OMNI scale Isometric resistance exercise: 6 to 8 on the OMNI scale	18 weeks/18th week
Rahimi 2018	NRS	31.5 ± 7.8	Healthy non-athlete women *n* = 10	Aerobic exercise	60% to 80% of maximal HR	12 weeks/24 and 72 h after the last exercise session
Saghiv 2016	NRS	53 ± 1.9	Coronary artery disease and healthy subjects *n* = 160	Aerobic exercise	60% to 75% of work capacity	36 weeks/36th week
Saghiv 2017	NRS	60 ± 2.3	Coronary artery disease *n* = 58	Aerobic exercise	75% to 80% of maximal HR	12 weeks/12th week
Saghiv 2019	RCT	27 ± 1.1	Adult males *n* = 60	Group 1: low intensity aerobic exercise Group 2: high intensity aerobic exercise	Low intensity aerobic exercise: anaerobic threshold at 40–50% VO_2_max High intensity aerobic exercise: 65–70% VO_2_max	16 and 24 weeks/16th and 24th weeks
Sharaf 2019	RCT	50.3 ± 3.8	Overweight postmenopausal women *n* = 48	Pilates-based exercise	11 to 13 on the Borg scale	12 weeks/48 h after the last session
Silva-Reis 2022	NRS	30 to 59	Eutrophic, obese I and overweight adults *n* = 40	Aerobic and resistance exercise	Aerobic exercise: 70% to 80% of the maximal HR Resistance exercise: load increased weekly by 2 to 10%	12 weeks/24 h after the last session
Snigdha 2024	RCT	60 to 80	Sedentary older adults *n* = 220	Yoga-based exercise	Moderate-intensity yoga sessions	26 weeks/26th week
Vazquez-Lorente 2023	RCT	53.6 ± 5.1	Sedentary middle-aged adults *n* = 74	Group 1: aerobic and resistance exercise Group 2: short and long high intensity aerobic interval exercise Group 3: short and long aerobic interval exercise + WB-EMS	Aerobic and resistance exercise: 60% to 65% of reserve HR and 40% to 50% 1-RM Short and long aerobic interval exercise: high intensity; > 95% VO_2_ max and > 120% VO_2_ max Short and long aerobic interval exercise + WB-EMS: high intensity; 80 mA to 100 mA; duty cycle 50–99%	12 weeks/12th week
Werner 2019	RCT	49 ± 7.1	Healthy inactive subjects *n* = 124	Group 1: aerobic exercise Group 2: aerobic interval exercise Group 3: resistance exercise	Aerobic exercise: 60% of reserve HR Aerobic interval exercise: high intensity; 4 × 4 method Resistance exercise: adjustment based on 20-RM	24 weeks/24th week

Abbreviations: *NRS* Non-randomized study; *RCT* Randomized controlled trial; *WB-EMS* Whole-body electromyostimulation; *HR* Heart rate; *RPE* Rate of perceived exertion; *MET* Metabolic equivalente of task; *1-RM* One-repetition maximum

#### Acute exercise

Thirteen studies investigated the impact of single-day exercise on s-Klotho levels [1, 18, 25, 32, 35, 56–59, 66, 73, 84, 85]. Four studies performed the Bruce protocol treadmill test [32, 66, 73, 84], 1 applied the Chester incremental step test [1], 1 used the Åstrand-Ryhming test on a cyclergometer [85], 2 completed a personalized incremental aerobic tests [25, 59], 3 conducted a personalized aerobic exercise session of moderate and constant intensity [18, 35, 57], 2 used a constant moderate-to-high intensity resistance exercise session [56, 57], 1 combined low-to-moderate-intensity aerobic exercise with high-intensity resistance exercise [57], and 1 performed a physical conditioning session [58]. In most of the studies, exercise was prescribed at moderate-to-high intensity, based on the percentage of maximum heart rate (%HR_max_) or percentage of maximum oxygen consumption (%VO_2max_) for aerobic exercise, and on one-repetition maximum (1-RM) and rate of perceived exertion (RPE) for resistance exercise.

#### Subacute exercise

The duration of these studies ranged from 2 to 8 weeks and were performed 3 to 5 times a week in 7 studies [24, 43, 54, 64, 67, 68, 71]. All of them prescribed aerobic exercise of mostly moderate-to-high intensity based on percentages of age-predicted %HR_max_, percentages of heart rate reserve (%HRR) or %VO_2max_ [24, 43, 54, 64, 67, 68, 71] and one prescribed moderate-to-high intensity resistance exercise based on %1-RM [24].

#### Chronic exercise

Twenty-one studies evaluated the impact of chronic exercise on s-Klotho levels [3, 6, 13, 14, 16, 17, 19, 23, 53, 55, 60, 61, 66, 71, 72, 74, 78, 80, 81, 89, 92]. They ranged from 12 to 36 weeks, with sessions conducted 2 to 5 days/week. Most studies implemented a weekly frequency of 3 sessions [3, 6, 14, 17, 19, 23, 55, 60, 61, 66, 78, 80, 89, 92]. Twelve studies prescribed moderate-to-high-intensity aerobic exercise based on percentages of %HRR, %HR_max_, and %VO_2max_ [3, 13, 23, 53, 55, 60, 66, 71, 72, 74, 89, 92]. Nine studies performed moderate-to-high-intensity resistance exercise based on %1-RM, %20-RM and RPE [9, 14, 16, 17, 49, 61, 78, 81, 92]. Six studies implemented the combination of aerobic and resistance exercise [3, 6, 19, 60, 80, 89], mostly at moderate intensity. Aerobic exercise was prescribed based on %HRR, %HR_max_ and RPE, and resistance exercise was prescribed based on %1-RM and RPE.

### Meta-analysis

#### Acute and subacute exercise

**Fig. 2:**
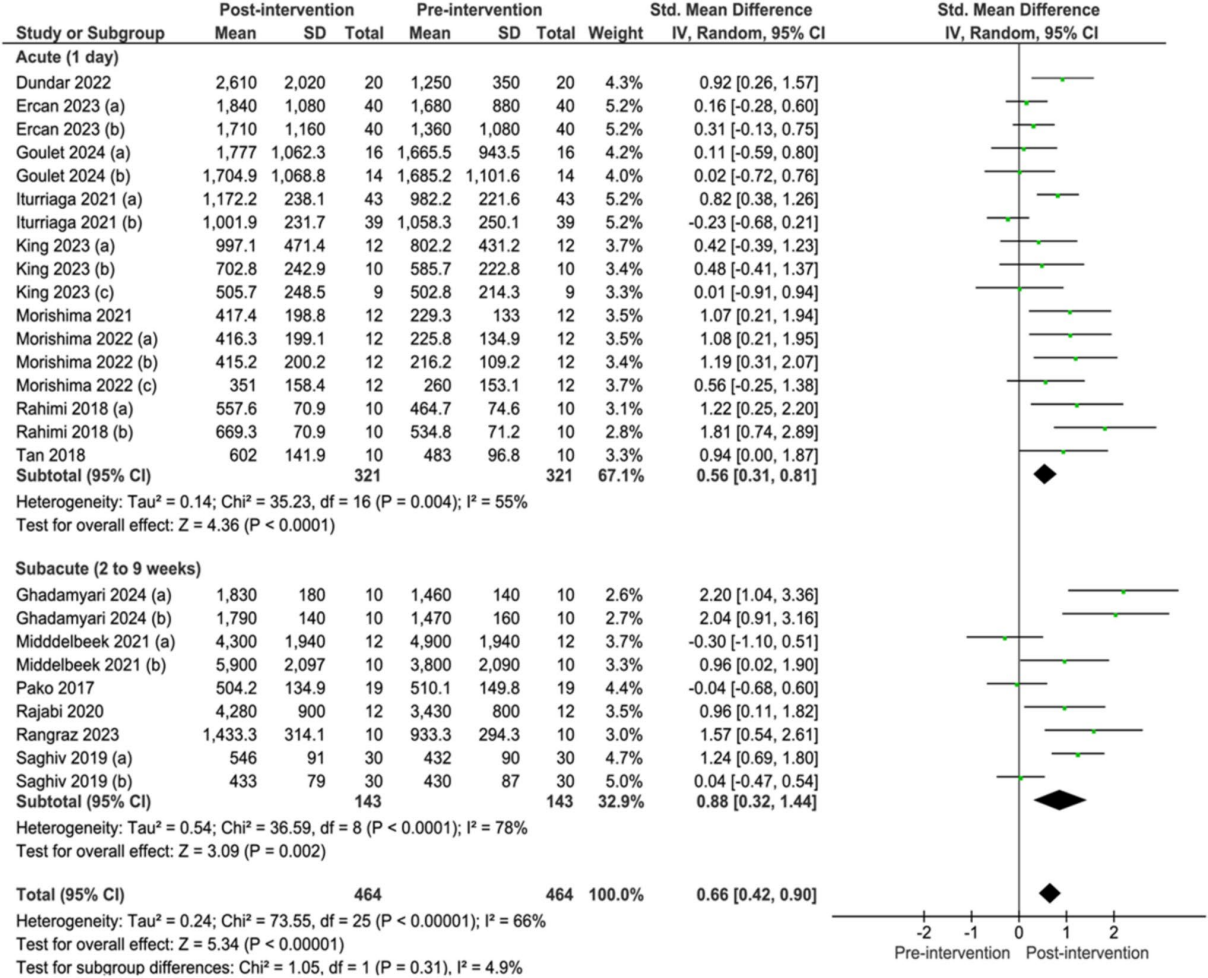
Forest plot of comparison: pre-intervention versus post-intervention; outcome: serum klotho concentration after acute and subacute exercise. SD = standardized deviation, STD = standardized, CI = confidence interval

Both single-day and subacute exercise significantly increased s-Klotho levels. Based on 9 studies [18, 25, 32, 35, 56, 57, 66, 84, 85], a single-day exercise session increased s-Klotho levels compared to pre-session timepoint (SMD 0.56; 95%CI 0.31 to 0.81; I^2^ = 55%; *p < *0.0001). Similarly, 6 studies [24, 54, 64, 67, 68, 71] reported that subacute exercise also increased s-Klotho levels compared to baseline (SMD 0.88; 95%CI 0.32 to 1.44; I^2^ = 78%; *p = *0.002) (Fig. 2).

##### Participant`s health status

Acute and subacute exercise significantly increased s-Klotho levels in healthy individuals and patients. Based on 12 studies [18, 25, 32, 35, 54, 56, 57, 66, 68, 71, 84, 85], healthy subjects exhibited a significant increase in s-Klotho levels compared to baseline (SMD 0.69; 95%CI 0.41 to 0.97; I^2^ = 65%; *p < *0.00001). Six studies [18, 24, 25, 35, 64, 67] reported significant changes in s-Klotho leves in patients compared to the baseline (SMD 0.62; 95%CI 0.11 to 1.12; I^2^ = 70%; *p = *0.02) (Supplementary Material 2). Specifically, 2 studies [24, 67] found that individuals with type II diabetes experienced significant alterations in s-Klotho levels with subacute exercise (SMD 1.65; 95%CI 0.84 to 2.47; I^2^ = 46%; *p < *0.0001), however, based on 2 studies [25, 35] no changes were observed with acute exercise (SMD 0.04; 95%CI −0.52 to 0.59; I^2^ = 0%; *p = *0.90) (Supplementary Material 3).

##### Exercise modality

Acute and subacute aerobic and concurrent exercise significantly increased s-Klotho levels, while resistance exercise showed no effect. Based on 14 studies [18, 24, 25, 32, 35, 54, 57, 64, 66–68, 71, 84, 85], aerobic exercise increased s-Klotho levels compared to baseline (SMD 0.60; 95%CI 0.36 to 0.85; I^2^ = 60%; *p < *0.00001), whereas no differences were noted in response to resistance exercise (SMD 0.95; 95%CI −0.10 to 2.00; I^2^ = 86%; *p = *0.08) [24, 32, 56, 57]. Additionally, one study [57] reported that acute concurrent exercise significantly increased s-Klotho levels compared to baseline (SMD 1.08; 95%CI 0.21 to 1.95; I^2^ = not applicable; *p = *0.01) (Supplementary Material 4).

##### Exercise frequency

Subacute exercise significantly increase s-Klotho levels compared to baseline at a exercise frequency of 3 sessions per week, while other exercise frequencies did not show significant changes. Based on 4 studies [24, 54, 67, 68], exercising three times per week showed a significant increase (SMD 1.18; 95%CI 0.42 to 1.94; I^2^ = 73%; *p = *0.002). A single study [71], reported no significant changes with four sessions per week (SMD 0.63; 95%CI −0.55 to 1.82; I^2^ = 90%; *p = *0.29). Similarly, one study [64], found no effect with five sessions per week (SMD −0.04; 95%CI −0.68 to 0.60; I^2^ = not applicable; *p = *0.90) (Supplementary Material 5).

#### Chronic exercise

**Fig. 3:**
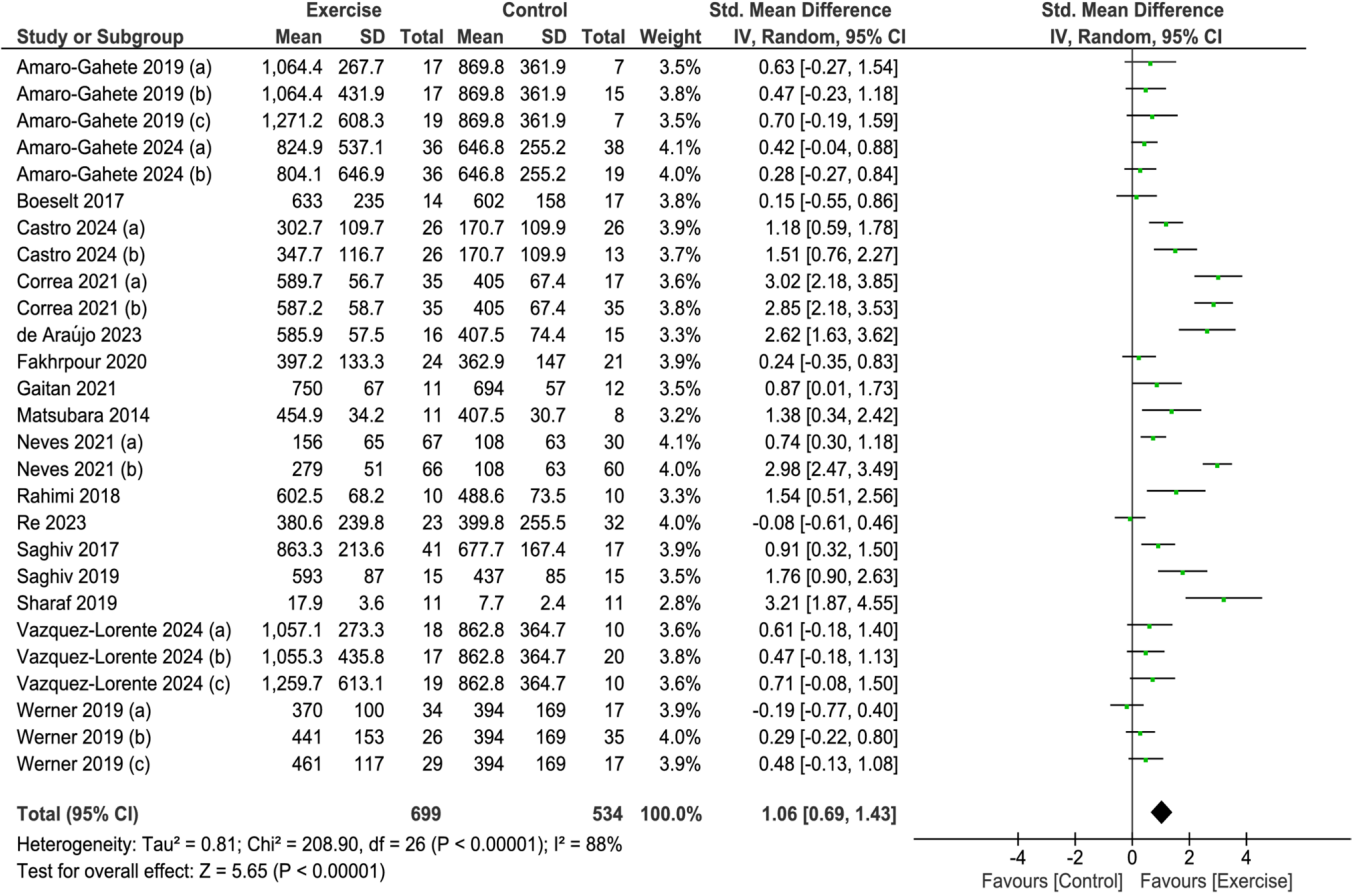
Forest plot of comparison: exercise versus control; outcome: serum klotho concentration after chronic exercise. SD = standardized deviation; STD = standardized; CI = confidence interval

Based on 17 studies [3, 6, 9, 14, 16, 17, 19, 23, 49, 53, 61, 66, 71, 72, 78, 89, 92], chronic exercise significantly increased s-Klotho levels compared to the controls (SMD 1.06; 95%CI 0.69 to 1.43; I^2^ = 88%; *p < *0.00001) (Fig. 3).

##### Participant’s health status

Chronic exercise significantly increased s-Klotho levels compared to controls, regardless of health status. Based on 7 studies [3, 6, 53, 66, 71, 89, 92], healthy individuals showed a significant increase (SMD 0.57; 95%CI 0.33 to 0.82; I^2^ = 42%; *p < *0.00001). Similarly, 10 studies [9, 14, 16, 17, 19, 23, 49, 61, 72, 78] reported an increase in diseased subjects (SMD 1.51; 95%CI 0.87 to 2.16; I^2^ = 92%; *p < *0.00001) (Supplementary Material 6). Specifically, 5 studies [14, 16, 17, 19, 61] found that individuals with chronic kidney disease exhibited a significant increase in s-Klotho levels (SMD 1.87; 95%CI 1.06 to 2.68; I^2^ = 92%; *p < *0.00001) (Supplementary Material 7).

##### Exercise modality

Chronic exercise significantly increased s-Klotho levels compared to controls, regardless of the exercise modality. Based on 8 studies [3, 23, 53, 66, 71, 72, 89, 92], aerobic exercise led to a significant increase (SMD 0.76; 95%CI 0.52 to 1.00; I^2^ = 8%; *p < *0.00001). Similarly, 8 studies [9, 14, 16, 17, 49, 61, 78, 92] reported a significant increase with resistance exercise (SMD 1.60; 95%CI 0.81 to 2.38; I^2^ = 94%; *p < *0.0001). Additionally, 4 studies [3, 6, 19, 89] found that concurrent exercise (aerobic and resistance) also increased s-Klotho levels (SMD 0.37; 95%CI 0.12 to 0.63; I^2^ = 0%; *p = *0.004) (Supplementary Material 8).

##### Exercise frequency

Chronic exercise increased s-Klotho levels compared to controls, with effects varying by weekly frequency. Based on 3 studies [3, 9, 89], exercising twice a week led to a significant increase (SMD 0.56; 95%CI 0.23 to 0.89; I^2^ = 0%; *p = *0.0008). Similarly, 9 studies [3, 14, 17, 19, 23, 61, 78, 89, 92] reported a greater increase with three weekly sessions (SMD 1.26; 95%CI 0.65 to 1.86; I^2^ = 92%; *p < *0.0001). Four studies [16, 53, 71, 72] also found a significant effect with four sessions per week (SMD 1.18; 95%CI 0.18 to 2.18; I^2^ = 84%; *p = *0.02). However, one study [49] reported that exercise five times per week did not increase s-Klotho levels (SMD −0.08; 95%CI −0.61 to 0.46; I^2^ = not applicable; *p = *0.78) (Supplementary Material 9).

### Risk of bias

#### Randomized controlled trials

**Fig. 4:**
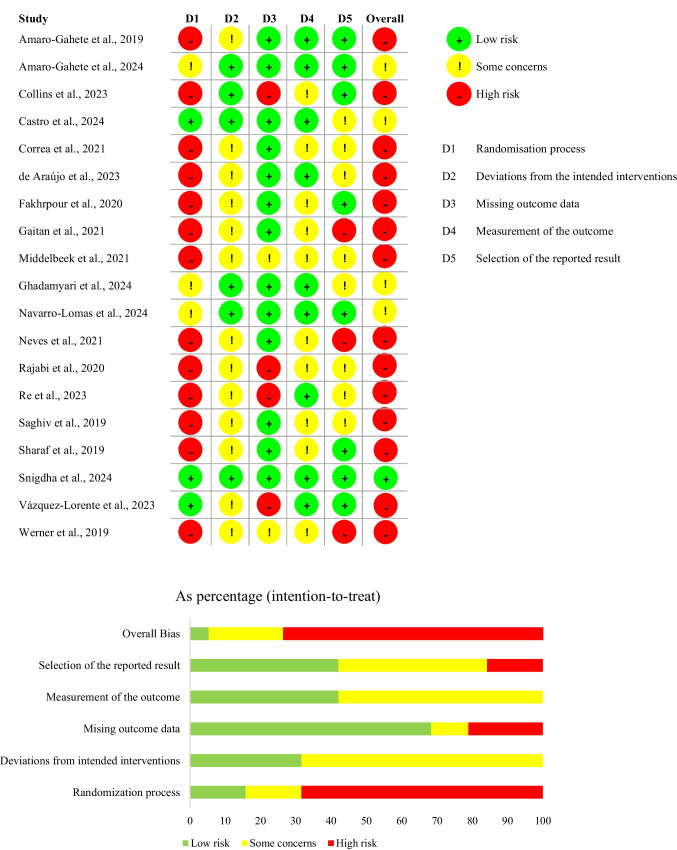
Assessment of the methodological quality of randomized controlled trials with the RoB 2 tool

The majority of RCTs showed a high risk of bias in the randomization process, excepting six studies [6, 17, 24, 60, 81, 89]. Thus, the overall risk of bias in most of the studies was classified as high. A proper methodological quality was observed in the domain of missing outcome data. (Fig. 4).

#### Non-randomized studies

**Fig. 5:**
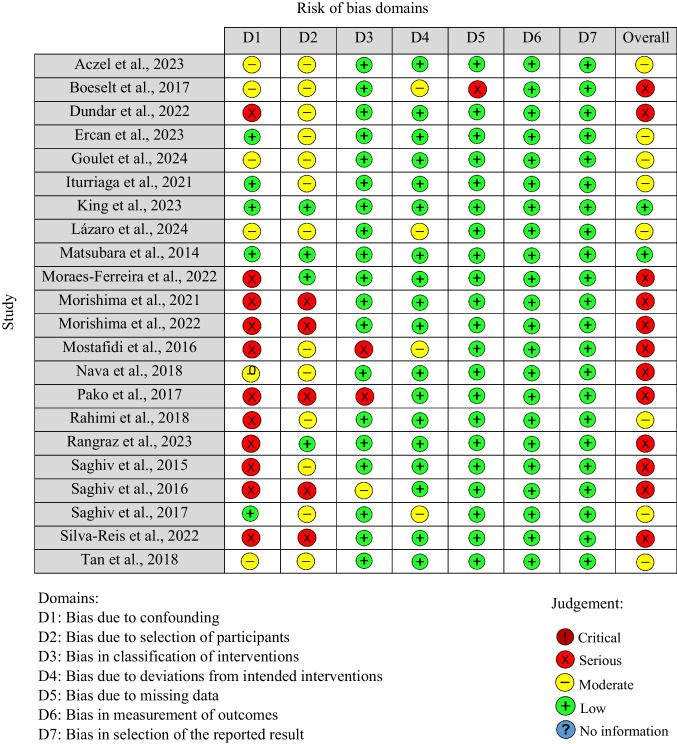
Assessment of the methodological quality of non-randomized studies with the ROBINS-I tool

Most of the NRSs exhibited a high risk of bias due to confounding, which contributed to an overall high risk of bias. Conversely, high methodological quality was noted in the domains of classification of interventions, deviations from intended interventions, missing data, measurement of outcomes, and selection of the reported result in the majority of the studies (Fig. 5).

### Quality of evidence

The quality of evidence was downgraded for most comparisons in the meta-analysis due to the overall presence of a high risk of bias, sample sizes below the optimal information size (n ≥ 400), and reliance on evidence from NRSs. Conversely, inconsistency was low, as indicated by low-to-moderate heterogeneity and considerable overlap of confidence intervals between studies. There was no indirectness for any comparison in the meta-analysis, while a strong suspicion of publication bias in five meta-analysis (Supplementary Material 10). Consequently, the quality of evidence ranged from very low to moderate (Supplementary Material 11).

## Discussion

The current results suggest that exercise plays an important role in modulating s-Klotho levels, with varying effects depending on the duration and exercise modality. In general, acute and subacute exercise induce a moderate increase in s-Klotho levels, with aerobic exercise being the primarily responsible for this effect. Acute to subacute exercise leads to a moderate increase in s-Klotho levels in healthy individuals, whereas no significant changes were observed in those with pathological conditions. However, chronic exercise demonstrated a greater impact, with a considerable increase in s-Klotho levels in both healthy people and in patients. Moreover, resistance exercise produced a positive large effect on s-Klotho levels, especially in patients, while chronic aerobic and concurrent exercise induced a moderate and small effects, respectively. Therefore, regular and prolonged practice of different exercise modalities seems to be essential for increasing s-Klotho, especially in a chronic context and among individuals with diseases.

Multiple interconnected biological mechanisms appear to be responsible for the exercise-induced increase in s-Klotho levels. Firstly, a potential upregulation has been suggested via alternative splicing and activation of secretases, which promote the release of s-Klotho [37]. Another possibility for activating secretases would be through the increase in growth hormone levels induced by exercise [22, 76]. Additionally, it has been shown that exercise-induced upregulation of the peroxisome proliferator-activated receptor and downregulation of angiotensin II may contribute to enhanced klotho gene expression [94, 95]. Another important mechanism proposed is the chronic effect of exercise in reducing oxidative stress [90, 96], through the increase of endogenous antioxidant enzymes (i.e., superoxide dismutase and glutathione peroxidase) that mitigate the impact of reactive oxygen species on s-Klotho [28, 79]. It has also been suggested an exercise-induced reduction of pro-inflammatory cytokines (IL-1) and the enhancement of anti-inflammatory cytokines (IL-10), which together may create a favorable environment for s-Klotho expression [48, 51, 68]. Moreover, exercise can also reduce levels of endothelin-1, which may improve endothelial function and elevate levels of s-Klotho [50, 56, 93]. Thus, exercise involves a combination of mechanisms that potentially may increase s-Klotho levels and promote cardiovascular and central nervous system health, while extending human´s longevity.

The effect of chronic exercise was greater compared with acute and subacute stimulus. This may be potentially explained due to repeated and continuous exposure to exercise, which induces physiological and metabolic adaptations that promotes a favorable hormonal environment for prolonged s-Klotho expression [36, 66, 69, 87]. Furthermore, it has been hypothesized that a longer exercise period may enhance communication between skeletal muscle and distant organs through the release of myokines [7, 8].

Chronic resistance exercise has exhibited the largest effect size in increasing s-Klotho levels, possibly due to the role of skeletal muscle as an active endocrine organ [29]. It has been demonstrated that, during contractions, skeletal muscle synthesizes and secretes several myokines which produce beneficial effects on peripheral and remote organs [31]. Myokines are released into the bloodstream, reaching target organs and acting in a paracrine manner within the muscle itself [65, 70]. In this context, Avin et al. (2014) [8] postulated that skeletal muscle contraction modulates klotho expression, which could explain the greater impact of resistance exercise. However, the authors pointed out that it is still unclear whether skeletal muscle contraction results directly in the local production and secretion of s-Klotho into the bloodstream or if a secreted myokine is responsible for inducing Klotho expression in other organs, such as the kidneys or brain [8].

Individuals with chronic diseases, as well as those with older ages, frequently experience exacerbated oxidative stress following acute exercise compared to the healthy controls [20, 21, 62]. On the other hand, exercising for a longer duration promotes a positive regulation of antioxidant defenses [38, 46, 90, 96]. This may partially explain why chronic exercise exhibited a larger effect size compared to acute or subacute exercise on the increase s-Klotho levels in patients with pathological conditions, as oxidative stress can regulate and be regulated by s-Klotho [26].

The weekly frequency of chronic exercise interventions exhibits a non-linear impact on s-Klotho levels, suggesting an inverted U-shaped relationship. These findings suggest an optimal frequency range, with three times a week producing the largest effect size for enhancing s-Klotho levels. In contrast, excessive frequency may be counterproductive, potentially due to factors such as overtraining or insufficient recovery time [33, 52]. Similarly, subacute exercise performed three times a week also produced a large effect size, but higher frequencies did not influence the increase in s-Klotho.

The limitations of this meta-analysis include the low methodological quality studies and the low certainty of evidence, which suggests that the conclusions should be interpreted with caution. Moreover, there is no standardization across the studies regarding the timing of blood collection for s-Klotho measurement after the intervention, or this information was not explicitly reported, which limits the determination of the most appropriate period for capturing variations in concentration, such as immediately, after 24 h, or later. Additionally, the pooled data from studies investigating acute and subacute exercise are based on pre- and post-intervention measurements within a single group, whereas the pooled data from studies investigating chronic exercise are derived from comparisons with a control group, which may introduce differences in the interpretation of results. Although circulating s-Klotho was predominantly quantified using commercial ELISA assays, most frequently from Immuno-Biological Laboratories (IBL), R&D Systems/Bio-Techne, and Demeditec, the majority of studies reported only the manufacturer and type of kit used, without providing detailed descriptions of additional analytical validation procedures. Therefore, in line with the concerns raised by Cheikhi et al. (2019) the heterogeneity of assays and the limited reporting of validation procedures may contribute to variability in Klotho measurements across studies and should be considered when interpreting the findings of this review [11]. On the other hand, strengths of this study include the subgroup analysis of the different forms of acute, subacute and chronic exercise in both healthy populations and patients, factors that expand the clinical applicability and external validity of the results. Furthermore, the searches were conducted extensively in the primary recommended databases and the quality of evidence was assessed using the GRADE approach.

Future investigations should explore the impact of combined exercise interventions on s-Klotho levels, as the effects of this modality of exercise are still not fully understood. Additionally, exploring the molecular mechanisms responsible for the exercise-induced increase in s-Klotho will contribute to the understanding of how different exercise types influence the regulatory pathways. The identification of s-Klotho as a potential biomarker for physical fitness should also be investigated, as this could provide fresh insights for clinical monitoring and personalized interventions. Finally, further studies are suggested to define the optimal frequency and intensity of exercise for both healthy populations and individuals with diseases, to modulate s-Klotho levels effectively, with the aim of influencing physiological processes and promoting health and longevity.

In summary, exercise leads to an increase in s-Klotho levels, with responses varying according to the modality and duration of exercise. Acute and subacute exercise, primarily aerobic and performed at moderate-to-high intensities, have been shown to elevate s-Klotho levels in healthy individuals and populations with underlying diseases. Chronic exercise interventions, ranging from 12 to 36 weeks, have demonstrated the greatest effect sizes, particularly when incorporating aerobic or resistance exercise performed three times per week at moderate-to-high intensities. This exercise modality has resulted in significant increases in s-Klotho levels in both healthy individuals and clinical populations, including those with chronic kidney disease. Despite these promising findings, methodological limitations, such as a high risk of bias and small sample sizes, compromise the quality of the evidence. Therefore, further well-designed, rigorous studies are required to confirm and strengthen these results.

## Supplementary Information

Below is the link to the electronic supplementary material.

Supplementary file1 (DOCX 30 KB)

Supplementary file2 (DOCX 805 KB)

Supplementary file3 (DOCX 315 KB)

Supplementary file4 (DOCX 755 KB)

Supplementary file5 (DOCX 696 KB)

Supplementary file6 (DOCX 837 KB)

Supplementary file7 (DOCX 408 KB)

Supplementary file8 (DOCX 749 KB)

Supplementary file9 (DOCX 672 KB)

Supplementary file10 (DOCX 1030 KB)

Supplementary file11 (DOCX 58 KB)

## Data Availability

No datasets were generated or analysed during the current study.
