# Supplementary material for: Effects of acute, subacute, and chronic exercise on plasma s-Klotho levels: a systematic review and meta-analysis
Source: J Physiol Biochem. 2026 May 2;82(1):46. doi: 10.1007/s13105-026-01182-2 (PMC13134988; doi:10.1007/s13105-026-01182-2)
Supplement: Supplementary file 1 — Supplementary file1 (DOCX 30 KB) [file 13105_2026_1182_MOESM1_ESM.docx]

Supplementary Material 1. Search strategy for each database.

**MEDLINE**

1. klotho.mp.
2. exp klotho proteins/
3. protein klotho.mp.
4. KL.mp.
5. KL1.mp.
6. S-klotho.mp.
7. S klotho.mp.
8. serum klotho.mp.
9. serum-klotho.mp.
10. soluble klotho.mp.
11. soluble-klotho.mp.
12. alpha-klotho.mp.
13. alpha klotho.mp.
14. alphaklotho.mp.
15. aKlotho.mp.
16. clotho.mp.
17. plasma klotho.mp.
18. circulating klotho.mp.
19. klotho peptide.mp.
20. klotho-related protein*.mp.
21. klotho level*.mp.
22. klotho plasma level*.mp.
23. plasma klotho level*.mp.
24. serum klotho level*.mp.
25. klotho serum level*.mp.
26. klotho concentration*.mp.
27. klotho gene.mp.
28. hypomorphic Klotho gene.mp.
29. klotho-related gene*.mp.
30. klotho hormone.mp.
31. hormone klotho.mp.
32. simply klotho.mp.
33. klotho measurement.mp.
34. klotho expression.mp.
35. FGF23.mp.
36. FGF23-klotho.mp.
37. fibroblast growth factor 23-Klotho.mp.
38. alphaKlotho-FGF.mp.
39. alpha-Klotho-FGF.mp.
40. klotho-derived peptide.mp.
41. klotho coreceptor.mp.
42. klotho co-receptor.mp.
43. fibroblast growth factor 23.mp.
44. fibroblast growth factor-23.mp.
45. FGF-FGFR.mp.
46. exerkine*.mp.
47. myokin*.mp.
48. grown hormone.mp.
49. GH-IGF-1 axis.mp.
50. insulin-like growth fator.mp.
51. or/1-50
52. exp physical therapy modalities/
53. exp exercise movement techniques/
54. exp exercise/
55. exp muscle strength/
56. exp body weight/
57. exp rehabilitation/
58. exp physical fitness/
59. exp physical endurance/
60. exp exercise therapy/
61. exercis*.ti,ab.
62. exercise therapy.mp.
63. endurance.ti,ab.
64. physical exercise.mp.
65. physical exertion.mp.
66. sport*.mp.
67. athletic*.mp.
68. swimming*.mp.
69. (bicycling* or cycling*).mp.
70. running*.mp.
71. (tai chi or tai ji or taiji or taijiquan).tw,kf.
72. (yoga or pilates).tw,kf.
73. (calisthenic$ or callisthenic$).tw,kf,ot.
74. (wb-ems or whole-body electromyostimulation).mp.
75. (whole body adj1 vibration*).mp.
76. (nmes or neuromuscular electrical stimulation).mp.
77. (electrical muscle stimulation or electrical stimulation or functional electrical stimulation).mp.
78. train*.mp.
79. activit*.mp.
80. (rehabilitat$ or fitness$ or exercis$ or physical$ or train$ or activ$ or physiotherap$ or kinesiotherap$ or exert$).ti,ab.
81. aerobic$.ti,ab.
82. treadmill.ti,ab.
83. strengthening program*.mp.
84. progressive resistance training.mp.
85. (muscle$ adj3 resist$).ti,ab.
86. (training adj1 program$).tw,kf,ot.
87. (muscular$ adj fitness$).tw,kf,ot.
88. exertion$.tw,kf,ot.
89. (resistanc$ adj2 (training$ or exercise$)).tw,kf,ot.
90. (muscular$ adj fitness$).tw,kf,ot.
91. (physical$ adj (activit$ or fitness$ or exercise$)).tw,kf,ot.
92. (physical$ adj (condition$ or effort4 or train$)).tw,kf,ot.
93. (aerobic$ adj train$).tw,kf,ot.
94. (human adj1 physical adj1 conditioning$).tw,kf,ot.
95. (resist$ training or strength$).tw.
96. ((strength$ or resist$ or weight$) adj3 training).tw.
97. or/52-96
98. (controlled clinical trial or randomized controlled trial).pt.
99. (randomized or randomised).ab,ti.
100. placebo.ab,ti.
101. randomly.ab,ti.
102. trial.ab,ti.
103. groups.ab,ti.
104. exp non-randomized controlled trials as topic/
105. exp controlled before-after studies/
106. (trial or multicenter or multi center or multicentre or multi centre).ti.
107. (intervention? or effect? or impact? or controlled or control group? or (before adj5 after) or (pre adj5 post) or ((pretest or pre test) and (posttest or post test)) or quasiexperiment* or quasi experiment* or evaluat*).ti,ab.
108. or/100-109
109. Animals.mp.
110. Humans.mp.
111. 109 not (109 and 110)
112. 108 not 111
113. 51 and 97 and 112

**Embase**

1. klotho.mp.
2. exp klotho proteins/
3. protein klotho.mp.
4. KL.mp.
5. KL1.mp.
6. S-klotho.mp.
7. S klotho.mp.
8. serum klotho.mp.
9. serum-klotho.mp.
10. soluble klotho.mp.
11. soluble-klotho.mp.
12. alpha-klotho.mp.
13. alpha klotho.mp.
14. alphaklotho.mp.
15. aKlotho.mp.
16. clotho.mp.
17. plasma klotho.mp.
18. circulating klotho.mp.
19. klotho peptide.mp.
20. klotho-related protein*.mp.
21. klotho level*.mp.
22. klotho plasma level*.mp.
23. plasma klotho level*.mp.
24. serum klotho level*.mp.
25. klotho serum level*.mp.
26. klotho concentration*.mp.
27. klotho gene.mp.
28. hypomorphic Klotho gene.mp.
29. klotho-related gene*.mp.
30. klotho hormone.mp.
31. hormone klotho.mp.
32. simply klotho.mp.
33. klotho measurement.mp.
34. klotho expression.mp.
35. FGF23.mp.
36. FGF23-klotho.mp.
37. fibroblast growth factor 23-Klotho.mp.
38. alphaKlotho/FGF.mp.
39. alpha-Klotho/FGF.mp.
40. klotho-derived peptide.mp.
41. klotho coreceptor.mp.
42. klotho co-receptor.mp.
43. fibroblast growth factor 23.mp.
44. fibroblast growth factor-23.mp.
45. FGF-FGFR.mp.
46. exerkine*.mp.
47. myokin*.mp.
48. grown hormone.mp.
49. GH/IGF-1 axis.mp.
50. insulin-like growth fator.mp.
51. or/1-50
52. exp physical therapy modalities/
53. exp exercise movement techniques/
54. exp exercise/
55. exp muscle strength/
56. exp body weight/
57. exp rehabilitation/
58. exp physical fitness/
59. exp physical endurance/
60. exp exercise therapy/
61. exercis*.ti,ab.
62. exercise therapy.mp.
63. endurance.ti,ab.
64. physical exercise.mp.
65. physical exertion.mp.
66. sport*.mp.
67. athletic*.mp.
68. swimming*.mp.
69. (bicycling* or cycling*).mp.
70. running*.mp.
71. (tai chi or tai ji or taiji or taijiquan).tw,kf.
72. (yoga or pilates).tw,kf.
73. (calisthenic$ or callisthenic$).tw,kf,ot.
74. (wb-ems or whole-body electromyostimulation).mp.
75. (whole body adj1 vibration*).mp.
76. (nmes or neuromuscular electrical stimulation).mp.
77. (electrical muscle stimulation or electrical stimulation or functional electrical stimulation).mp.
78. train*.mp.
79. activit*.mp.
80. (rehabilitat$ or fitness$ or exercis$ or physical$ or train$ or activ$ or physiotherap$ or kinesiotherap$ or exert$).ti,ab.
81. aerobic$.ti,ab.
82. treadmill.ti,ab.
83. strengthening program*.mp.
84. progressive resistance training.mp.
85. (muscle$ adj3 resist$).ti,ab.
86. (training adj1 program$).tw,kf,ot.
87. (muscular$ adj fitness$).tw,kf,ot.
88. exertion$.tw,kf,ot.
89. (resistanc$ adj2 (training$ or exercise$)).tw,kf,ot.
90. (muscular$ adj fitness$).tw,kf,ot.
91. (physical$ adj (activit$ or fitness$ or exercise$)).tw,kf,ot.
92. (physical$ adj (condition$ or effort4 or train$)).tw,kf,ot.
93. (aerobic$ adj train$).tw,kf,ot.
94. (human adj1 physical adj1 conditioning$).tw,kf,ot.
95. (resist$ training or strength$).tw.
96. ((strength$ or resist$ or weight$) adj3 training).tw.
97. or/52-96
98. (controlled clinical trial or randomized controlled trial).ab,ti.
99. (randomized or randomised).ab,ti.
100. placebo.ab,ti.
101. randomly.ab,ti.
102. trial.ab,ti.
103. groups.ab,ti.
104. exp non-randomized controlled trials as topic/
105. exp controlled before-after studies/
106. (trial or multicenter or multi center or multicentre or multi centre).ti.
107. (intervention? or effect? or impact? or controlled or control group? or (before adj5 after) or (pre adj5 post) or ((pretest or pre test) and (posttest or post test)) or quasiexperiment* or quasi experiment* or evaluat*).ti,ab.
108. or/98-107
109. 51 and 97 and 108

**CENTRAL**

1. MeSH descriptor: [Klotho Proteins] explode all trees
2. protein klotho
3. S-klotho
4. S klotho
5. serum klotho
6. serum-klotho
7. soluble klotho
8. soluble-klotho
9. alpha-klotho
10. alpha klotho
11. alphaklotho
12. aKlotho
13. clotho
14. plasma klotho
15. circulating klotho
16. klotho peptide
17. klotho-related protein*
18. klotho level*
19. klotho plasma level*
20. plasma klotho level*
21. serum klotho level*
22. klotho serum level*
23. klotho concentration*
24. klotho gene
25. hypomorphic Klotho gene
26. klotho-related gene*
27. klotho hormone
28. hormone klotho
29. simply klotho
30. klotho measurement
31. klotho expression
32. FGF23
33. FGF23-klotho
34. fibroblast growth factor 23-Klotho
35. alphaKlotho/FGF
36. alpha-Klotho/FGF
37. klotho-derived peptide
38. klotho coreceptor
39. klotho co-receptor
40. fibroblast growth factor 23
41. fibroblast growth factor-23
42. FGF-FGFR
43. exerkine*
44. myokin*
45. grown hormone
46. GH/IGF-1 axis
47. insulin-like growth fator
48. #1 OR #2 OR #3 OR #4 OR #5 OR #6 OR #7 OR #8 OR #9 OR #10 OR #11 OR #12 OR #13 OR #14 OR #15 OR #16 OR #17 OR #18 OR #19 OR #20 OR #21 OR #22 OR #23 OR #24 OR #25 OR #26 OR #27 OR #28 OR #29 OR #30 OR #31 OR #32 OR #33 OR #34 OR #35 OR #36 OR #37 OR #38 OR #39 OR #40 OR #41 OR #42 OR #43 OR #44 OR #45 OR #46 OR #47
49. MeSH descriptor: [Exercise] explode all trees
50. MeSH descriptor: [Physical Therapy Modalities] explode all trees
51. MeSH descriptor: [Rehabilitation] explode all trees
52. MeSH descriptor: [Physical Therapists] explode all trees
53. MeSH descriptor: [Physical Fitness] explode all trees
54. MeSH descriptor: [Physical Exertion] explode all trees
55. MeSH descriptor: [Physical Endurance] explode all trees
56. MeSH descriptor: [Exercise Therapy] explode all trees
57. MeSH descriptor: [Walking] explode all trees
58. MeSH descriptor: [Vibration] explode all trees
59. MeSH descriptor: [Electric Stimulation Therapy] explode all trees
60. MeSH descriptor: [Tai Ji] explode all trees
61. MeSH descriptor: [Dancing] explode all trees
62. MeSH descriptor: [Swimming] explode all trees
63. MeSH descriptor: [Yoga] explode all trees
64. MeSH descriptor: [Fitness Trackers] explode all trees
65. MeSH descriptor: [Sports] explode all trees
66. MeSH descriptor: [Running] explode all trees
67. Aerobic*
68. Resistance training
69. Resistance exercise
70. Neuromuscular electrical stimulation
71. NMES
72. Electrical muscle stimulation
73. Electrical stimulation
74. Functional electrical stimulation
75. Strength
76. #49 OR #50 OR #51 OR #52 OR #53 OR #54 OR #55 OR #56 OR #57 OR #58 OR #59 OR #60 OR #61 OR #62 OR #63 OR #64 OR #65 OR #66 OR #67 OR #68 OR #69 OR #70 OR #71 OR #72 OR #73 OR #74 OR #75
77. #48 AND #76

**CINAHL**

S1. (klotho) OR (klotho proteins) OR (protein klotho) OR (S-klotho) OR (S klotho) OR (serum klotho) OR (serum-klotho) OR (soluble klotho) OR (soluble-klotho) OR (α-klotho) OR (alpha-klotho) OR (alpha klotho) OR (alphaklotho) OR (alpha klotho) OR (aKlotho) OR (clotho) OR (plasma klotho) OR (circulating klotho) OR (klotho peptide) OR (klotho-related protein*) OR (klotho level*) OR (klotho plasma level*) OR (plasma klotho level*) OR (serum klotho level*) OR (klotho serum level*) OR (klotho concentration*) OR (klotho gene) OR (hypomorphic klotho gene) OR (klotho-related gene*) OR (klotho hormone) OR (hormone klotho) OR (simply klotho) OR (klotho measurement) OR (klotho expression.) OR (FGF23) OR (FGF23-klotho) OR (fibroblast growth factor 23-klotho) OR (alphaKlotho-FGF) OR (alpha-klotho-FGF) OR (klotho-derived peptide) OR (klotho coreceptor) OR (klotho co-receptor) OR (fibroblast grow factor 23) OR (fibroblast growth factor-23) OR (FGF-FGFR) OR (exerkine*) OR (myokin*) OR (grown hormone) OR (GH-IGF-1 axis) OR (insulin-like growth factor)

S2. (MH "movement+") OR (MH "physical therapy+") OR (MH "rehabilitation+") OR (MH "physical fitness+") OR (MH "muscle strength+") OR (MH "body weight+") OR (MH "exercise+") OR (MH "physical endurance+") OR (MH "pilates") OR (MH "yoga+") OR (MH “tai chi") OR (motor activity) OR (physical therapy) OR (exercise) OR (exercise movement) OR (muscle strength) OR (sport*) OR (aerobic*) OR (exercise therapy) OR (physical exercise) OR (physical exertion) OR (athletic*) OR (swimming*) OR (bicycling*) OR (cycling*) OR (running*) OR (calisthenic*) OR (wb-ems) OR (whole-body electromyostimulation) OR (whole body vibration) OR (nmes) OR (neuromuscular electrical stimulation) OR (electrical muscle stimulation) OR (electrical stimulation) OR (funcional electrical stimulation) OR (train*) OR (activit*) OR (physiotherapy) OR (kinesiotherapy) OR (treadmill) OR (strengthening program*) OR (resistance training) OR (progressive resistance training) OR (resistance exercise) OR (exertion) OR (physical activity) OR (physical conditioning) OR (strength)

S3. (randomized) OR (randomised) OR (treatment outcome*) OR (clinical trial*) OR (trial) OR (randomized controlled trial) OR (placebo) OR (groups) OR (non-randomized controlled trial*) OR (non-randomized) OR (controlled before-after study) OR (pre test) OR (post test) OR (quase experiment*)

S4. S1 AND S2 AND S3

**Scopus**

((TITLE-ABS-KEY (klotho OR “protein klotho” OR “myokine” OR “exerkine” OR “S-klotho” OR “soluble klotho” OR “serum klotho” OR “α-klotho” OR “alpha-klotho” OR aKlotho OR clotho OR “plasma klotho” OR “circulating klotho” OR “klotho peptide” OR “klotho plasma level” OR “klotho serum level” OR “klotho concentration” OR “klotho gene” OR “klotho hormone” OR “klotho measurement” OR “FGF-23” OR “FGF-23-klotho” OR “klotho-derived peptide” OR “klotho coreceptor” OR “GH-IGF-axis” OR “insulin-like growth fator”) AND TITLE-ABS-KEY (exercise OR “resistance training” OR “resistance exercise” OR “strength exercise” OR “progressive resistance training” OR aerobic OR “aerobic exercise” OR “aerobic training” OR “muscle strength” OR “body weight” OR “physical fitness” OR “exercise therapy” OR sport OR athletic OR swimming OR bicycling OR cycling OR running OR “tai chi” OR “tai ji” OR yoga OR pilates OR calisthenic OR “wb-ems” OR “whole-body electromyostimulation” OR “whole-body vibration” OR NMES OR “neuromuscular electrical stimulation” OR “electrical muscle stimulation” OR “electrical stimulation” OR “functional electrical stimulation” OR training OR physiotherapy OR rehabilitation OR treadmill OR “physical exertion” OR endurance OR “training program” OR “physical conditioning”) AND TITLE-ABS-KEY (“randomized controlled trial” OR RCT OR “controlled clinical trial” OR randomized OR randomised OR placebo OR randomly OR trial OR groups OR “quasi-randomized controlled trial” OR “quasi-experimental” OR “non-randomized” OR “non-randomized controlled trial”))

**Web of Science**

(klotho) OR (protein klotho) OR (myokine) OR (exerkine) OR (S-klotho) OR (soluble klotho) OR (serum klotho) OR (α-klotho) OR (alpha-klotho) OR (arlotto) OR (cloths) OR (plasma klotho) OR (circulating klotho) OR (klotho peptide) OR (klotho plasma level) OR (klotho serum level) OR (klotho concentration) OR (klotho gene) OR (klotho hormone) OR (klotho measurement) OR (FGF-23) OR (FGF-23-klotho) OR (klotho-derived peptide) OR (klotho coreceptor) OR (GH-IGF-axis) OR (insulin-like growth factor) (All Fields) AND (exercise) OR (resistance training) OR (resistance exercise) OR (strength exercise) OR (progressive resistance training) OR (aerobic) OR (aerobic exercise) OR (aerobic training) OR (muscle strength) OR (body weight) OR (physical fitness) OR (exercise therapy) OR (sport) OR (athletic) OR (swimming) OR (bicycling) OR (cycling) OR (running) OR (tai chi) OR (tai ji) OR (yoga) OR (pilates) OR (calisthenic) OR (wb-ems) OR (whole-body electromyostimulation) OR (whole-body vibration) OR (NMES) OR (neuromuscular electrical stimulation) OR (electrical muscle stimulation) OR (electrical stimulation) OR (functional electrical stimulation) OR (training) OR (physiotherapy) OR (rehabilitation) OR (treadmill) OR (physical exertion) OR (endurance) OR (training program) OR (physical conditioning) (Abstract) AND (randomized controlled trial) OR (RCT) OR (controlled clinical trial) OR (randomized) OR (randomised) OR (placebo) OR (randomly) OR (trial) OR (groups) OR (quasi-randomized controlled trial) OR (quasi-experimental) OR (non-randomized) OR (non-randomized controlled trial) (Abstract)

**LILACS**

((klotho) OR (protein klotho) OR (myokine) OR (exerkine) OR (S-klotho) OR (soluble klotho) OR (serum klotho) OR (α-klotho) OR (alpha-klotho) OR (aKlotho) OR (clotho) OR (plasma klotho) OR (circulating klotho) OR (klotho peptide) OR (klotho plasma level) OR (klotho serum level) OR (klotho concentration) OR (klotho gene) OR (klotho hormone) OR (klotho measurement) OR (FGF-23) OR (FGF-23-klotho) OR (klotho-derived peptide) OR (klotho coreceptor) OR (GH-IGF-axis) OR (insulin-like growth fator)) AND ((exercise) OR (resistance training) OR (resistance exercise) OR (strength exercise) OR (progressive resistance training) OR (aerobic) OR (aerobic exercise) OR (aerobic training) OR (muscle strength) OR (body weight) OR (physical fitness) OR (exercise therapy) OR (sport) OR (athletic) OR (swimming) OR (bicycling) OR (cycling) OR (running) OR (tai chi) OR (tai ji) OR (yoga) OR (pilates) OR (calisthenic) OR (wb-ems) OR (whole-body electromyostimulation) OR (whole-body vibration) OR (NMES) OR (neuromuscular electrical stimulation) OR (electrical muscle stimulation) OR (electrical stimulation) OR (functional electrical stimulation) OR (training) OR (physiotherapy) OR (rehabilitation) OR (treadmill) OR (physical exertion) OR (endurance) OR (training program) OR (physical conditioning)) AND ((randomized controlled trial) OR (RCT) OR (controlled clinical trial) OR (randomized) OR (randomised) OR (placebo) OR (randomly) OR (trial) OR (groups) OR (quasi-randomized controlled trial) OR (quasi-experimental) OR (non-randomized) OR (non-randomized controlled trial))

**SciELO**

((klotho) OR (protein klotho) OR (myokine) OR (exerkine) OR (S-klotho) OR (soluble klotho) OR (serum klotho) OR (α-klotho) OR (alpha-klotho) OR (aKlotho) OR (clotho) OR (plasma klotho) OR (circulating klotho)
