# Supplementary material for: Effects of acute, subacute, and chronic exercise on plasma s-Klotho levels: a systematic review and meta-analysis
Source: J Physiol Biochem. 2026 May 2;82(1):46. doi: 10.1007/s13105-026-01182-2 (PMC13134988; doi:10.1007/s13105-026-01182-2)
Supplement: Supplementary file 3 — Supplementary file3 (DOCX 315 KB) [file 13105_2026_1182_MOESM3_ESM.docx]

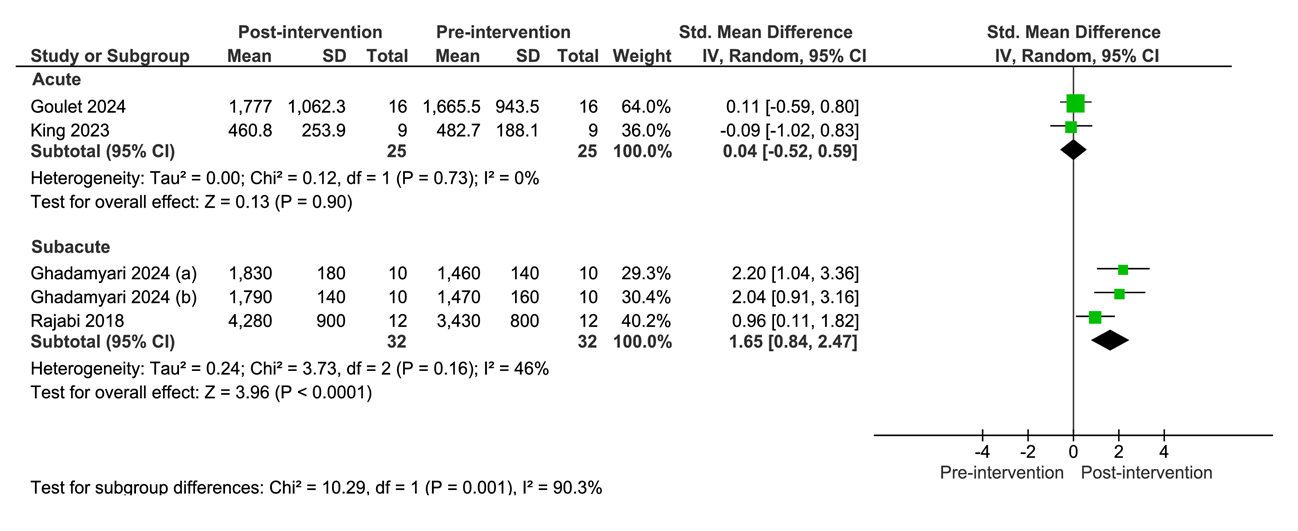


Supplementary Material 3. Forest plot of comparison: pre-intervention versus post-intervention; outcome: serum klotho concentration after acute /subacute exercise on subjects with type II diabetes. SD = standardized deviation; STD = standardized; CI = confidence interval.
