# Supplementary material for: Effects of acute, subacute, and chronic exercise on plasma s-Klotho levels: a systematic review and meta-analysis
Source: J Physiol Biochem. 2026 May 2;82(1):46. doi: 10.1007/s13105-026-01182-2 (PMC13134988; doi:10.1007/s13105-026-01182-2)
Supplement: Supplementary file 9 — Supplementary file9 (DOCX 672 KB) [file 13105_2026_1182_MOESM9_ESM.docx]

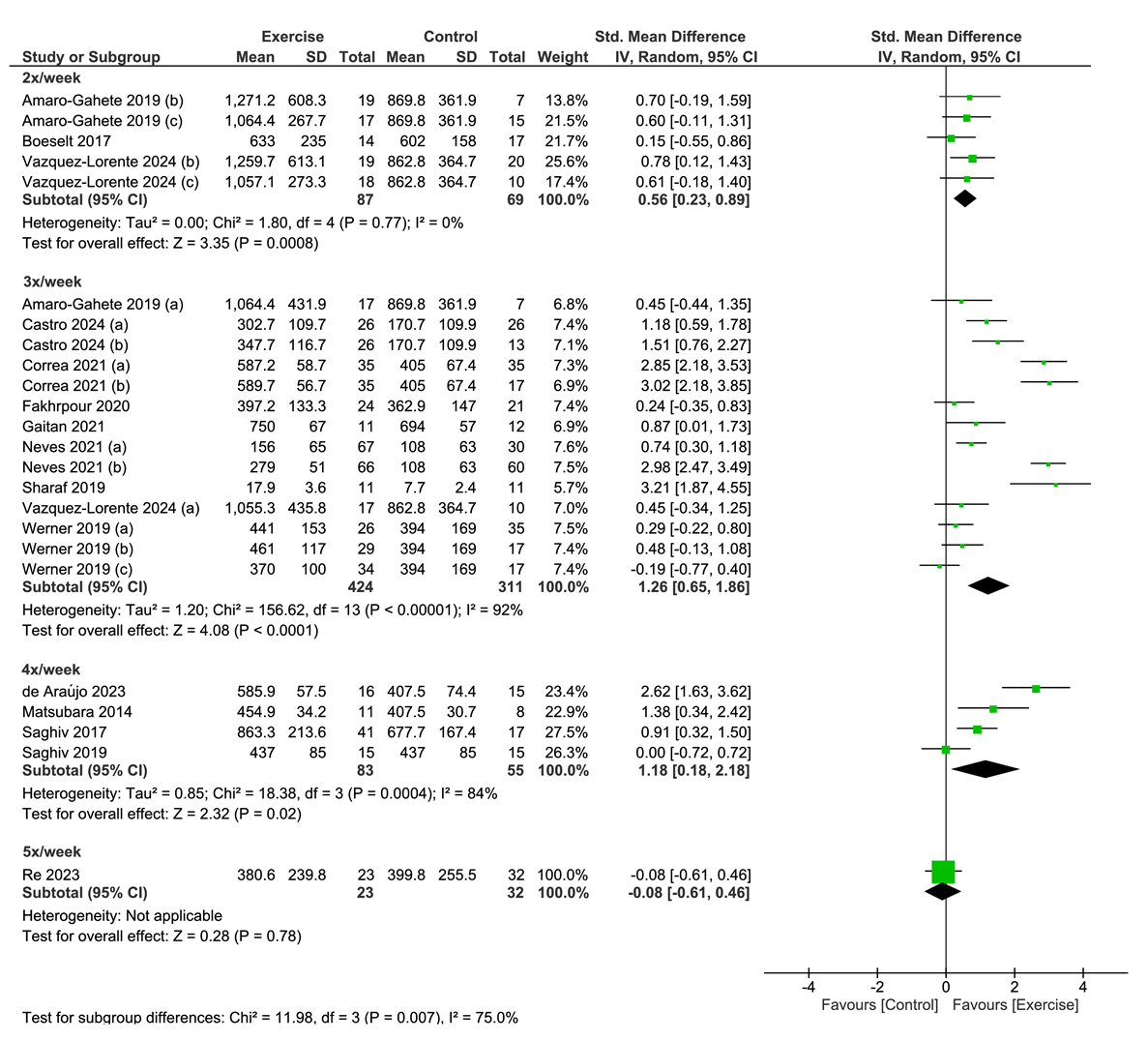


Supplementary Material 9. Forest plot of comparison: exercise versus control; outcome: serum klotho concentration after chronic exercise performed 2, 3, 4 or 5 times per week. SD = standardized deviation; STD = standardized; CI = confidence interval.
