# Supplementary material for: Effects of acute, subacute, and chronic exercise on plasma s-Klotho levels: a systematic review and meta-analysis
Source: J Physiol Biochem. 2026 May 2;82(1):46. doi: 10.1007/s13105-026-01182-2 (PMC13134988; doi:10.1007/s13105-026-01182-2)
Supplement: Supplementary file 10 — Supplementary file10 (DOCX 1030 KB) [file 13105_2026_1182_MOESM10_ESM.docx]

Supplementary Material 10 (A-G). Publication bias analysis.


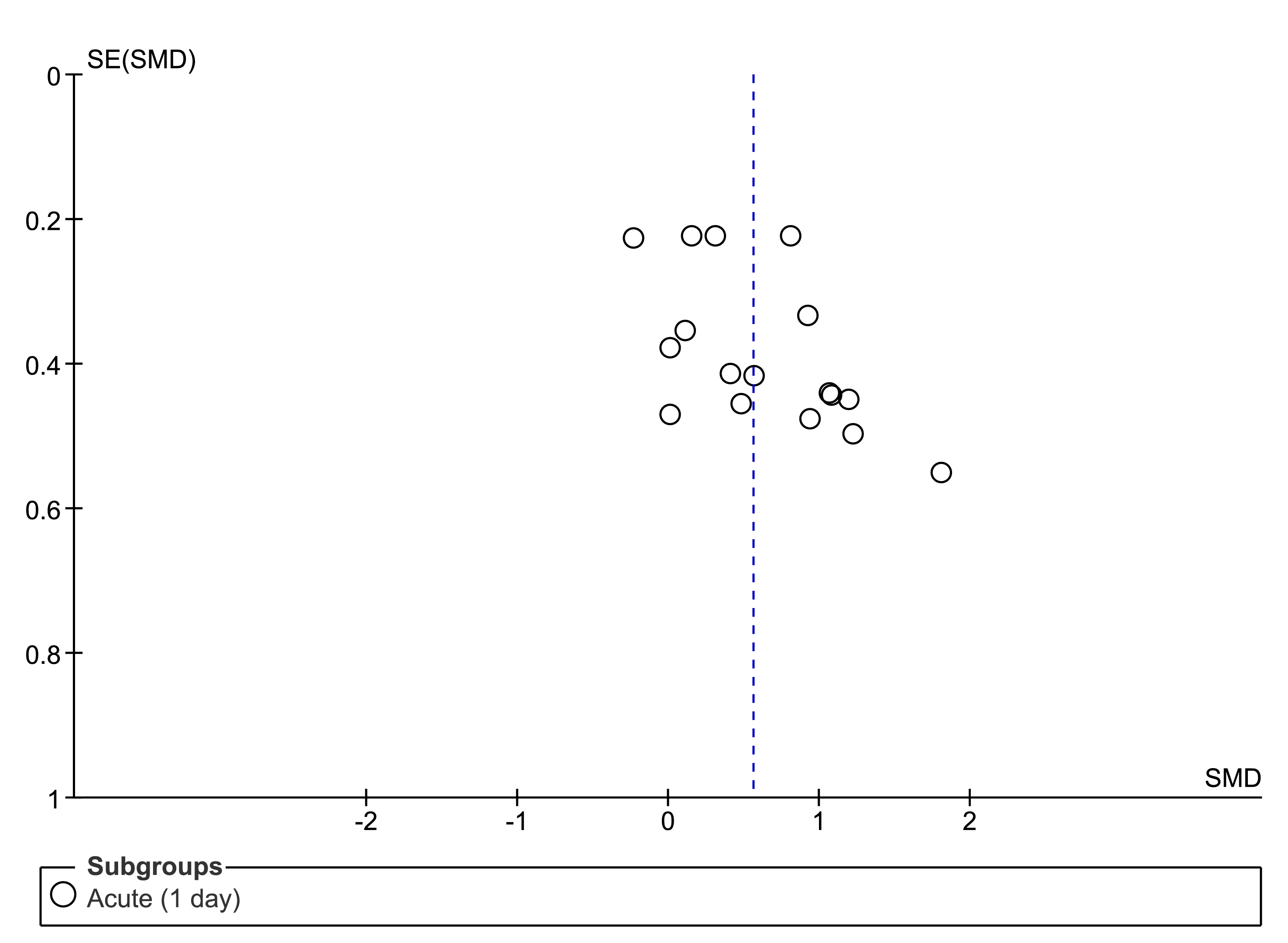


Supplementary Material 10A. Funnel plot of comparison: pre-intervention versus post-intervention; outcome: serum klotho concentration after acute exercise.

**Regression-based Egger test for small-study effects**

Random-effects model

Method: DerSimonian–Laird

H0: beta1 = 0; no small-study effects

beta1 = 4.35

SE of beta1 = 2.374

t = 1.83

Prob > |t| = 0.0869


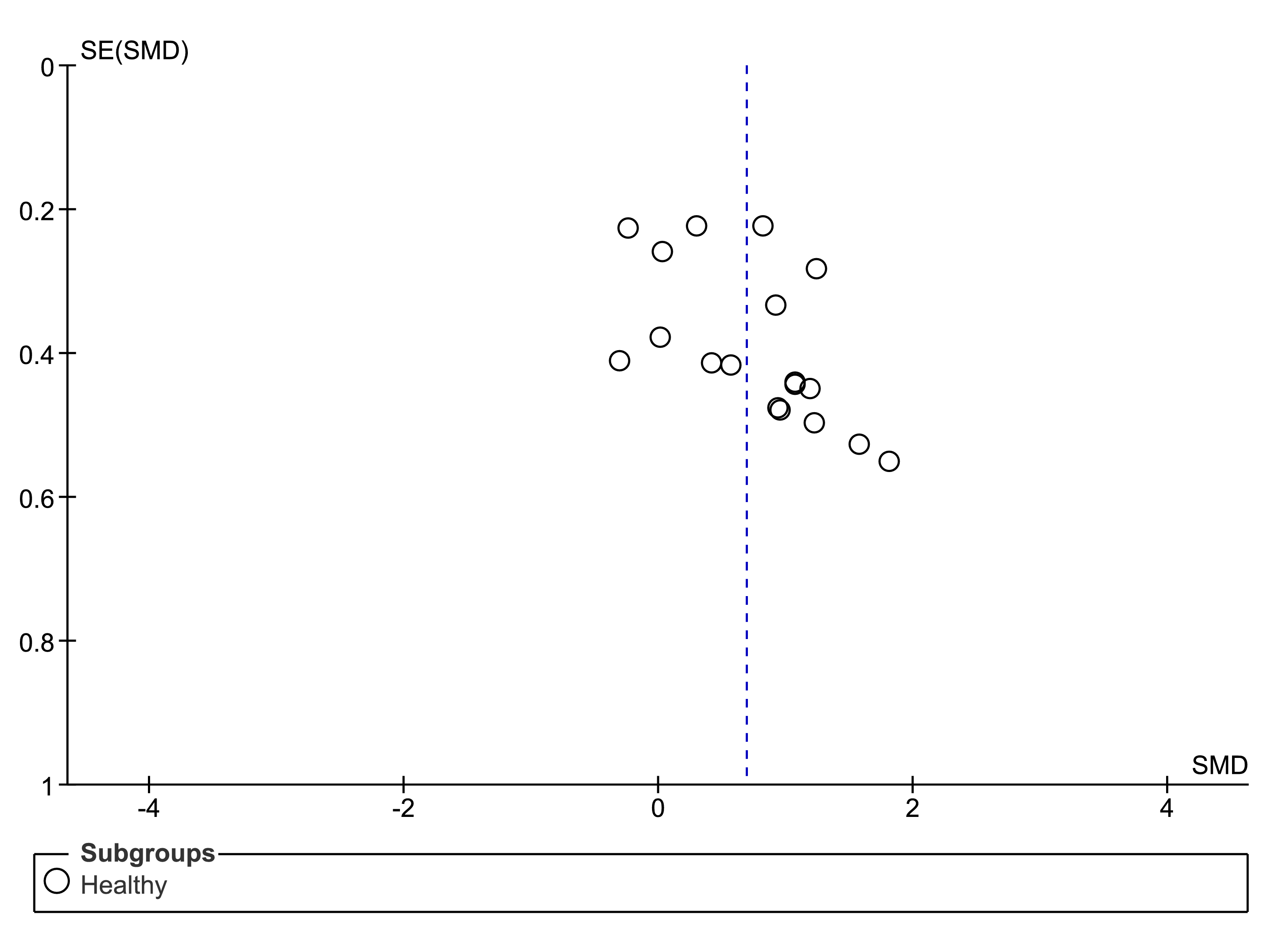


Supplementary Material 10B. Funnel plot of comparison: pre-intervention versus post-intervention; outcome: serum klotho concentration in healthy subjects after acute and subacute exercise.

**Regression-based Egger test for small-study effects**

Random-effects model

Method: DerSimonian–Laird

H0: beta1 = 0; no small-study effects

beta1 = 3.28

SE of beta1 = 1.316

t = 2.49

Prob > |t| = 0.0240*

* |t| ≤ 0.05 = statistically significant


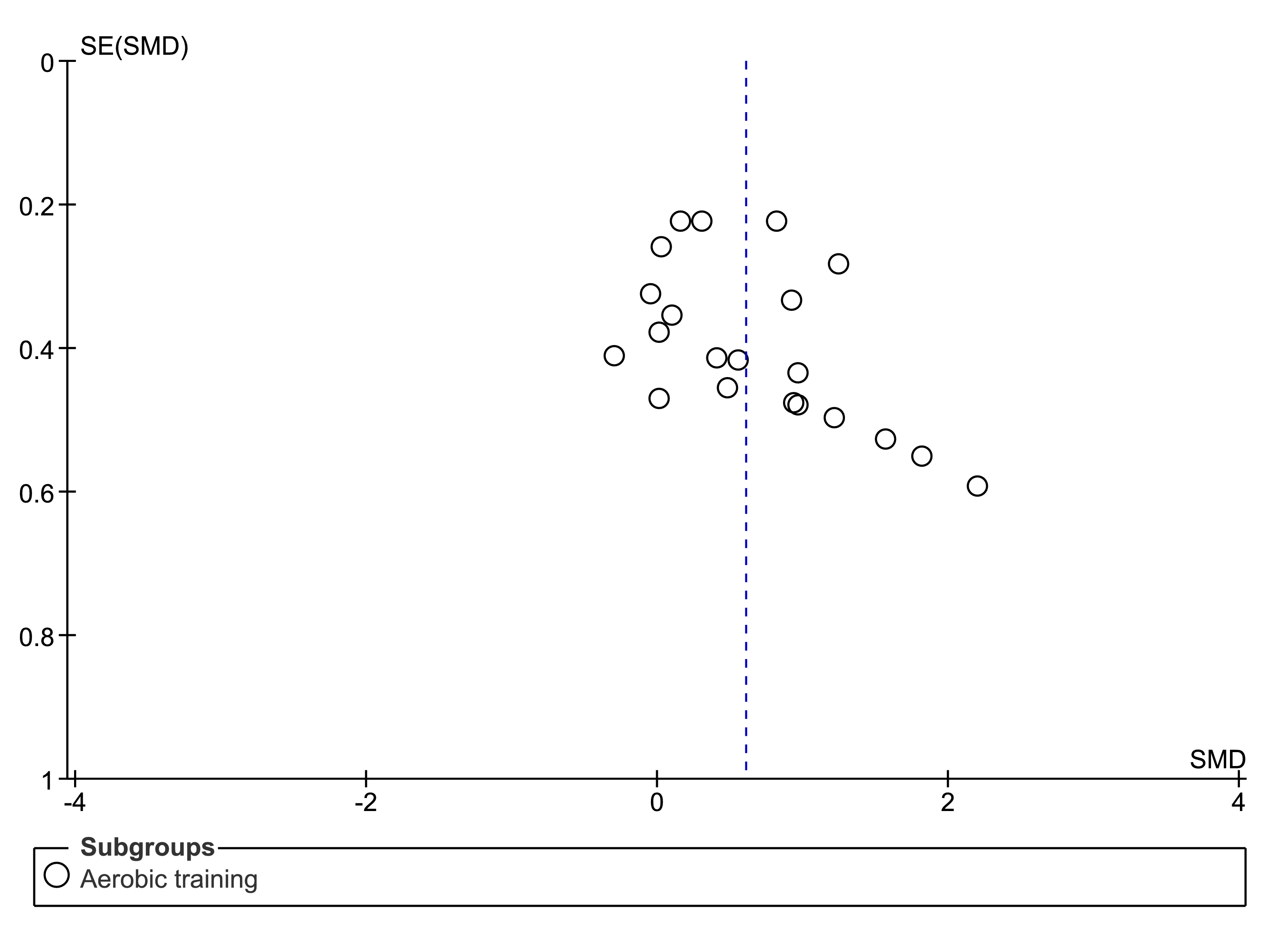


Supplementary Material 10C. Funnel plot of comparison: pre-intervention versus post-intervention; outcome: serum klotho concentration after acute and subacute aerobic exercise.

**Regression-based Egger test for small-study effects**

Random-effects model

Method: DerSimonian–Laird

H0: beta1 = 0; no small-study effects

beta1 = 2.95

SE of beta1 = 1.221

t = 2.42

Prob > |t| = 0.0258*

* |t| ≤ 0.05 = statistically significant


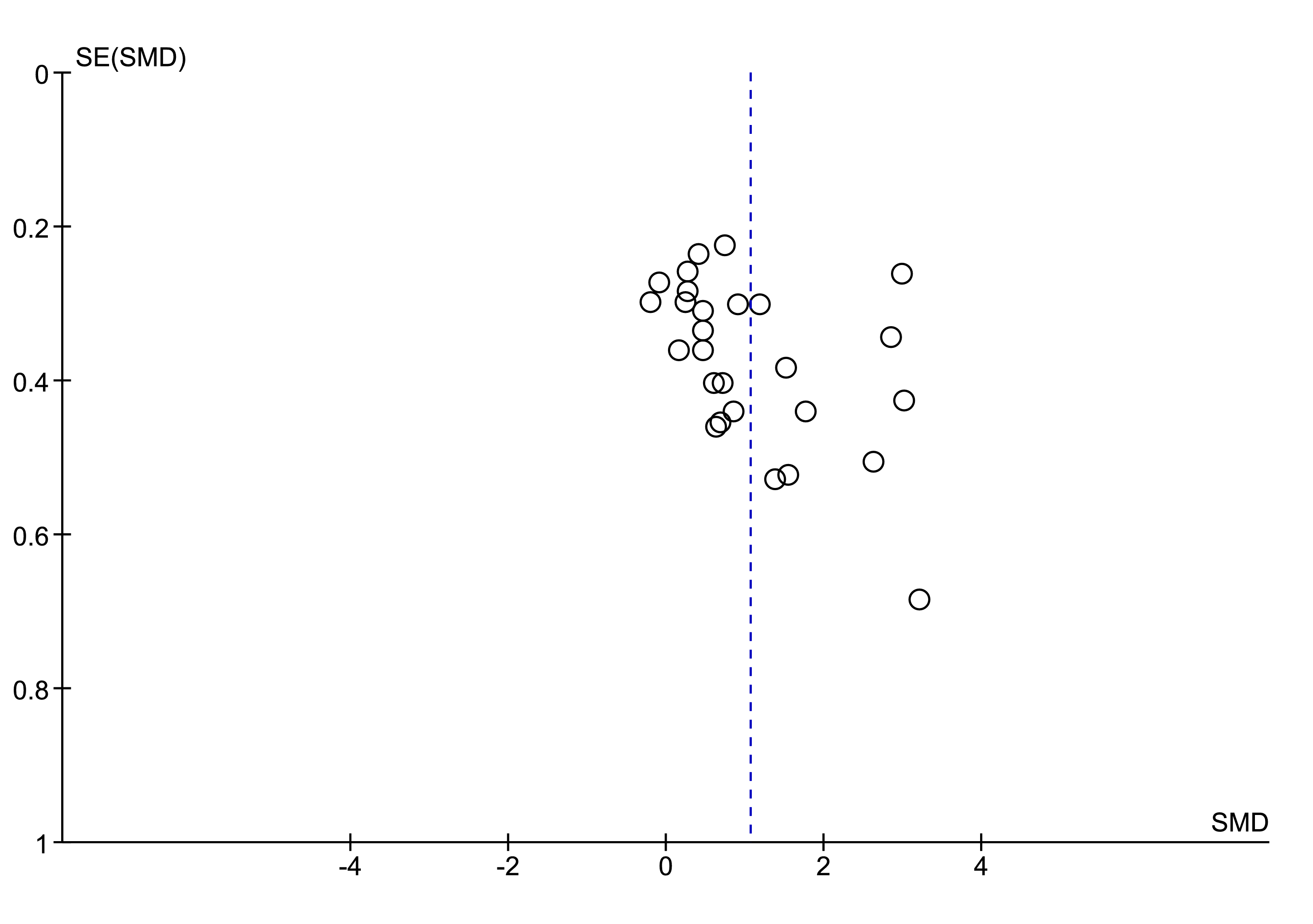


Supplementary Material 10D. Funnel plot of comparison: exercise versus control; outcome: serum klotho concentration after chronic exercise.

**Regression-based Egger test for small-study effects**

Random-effects model

Method: DerSimonian–Laird

H0: beta1 = 0; no small-study effects

beta1 = 5.01

SE of beta1 = 1.958

t = 2.56

Prob > |t| = 0.0169*

* |t| ≤ 0.05 = statistically significant


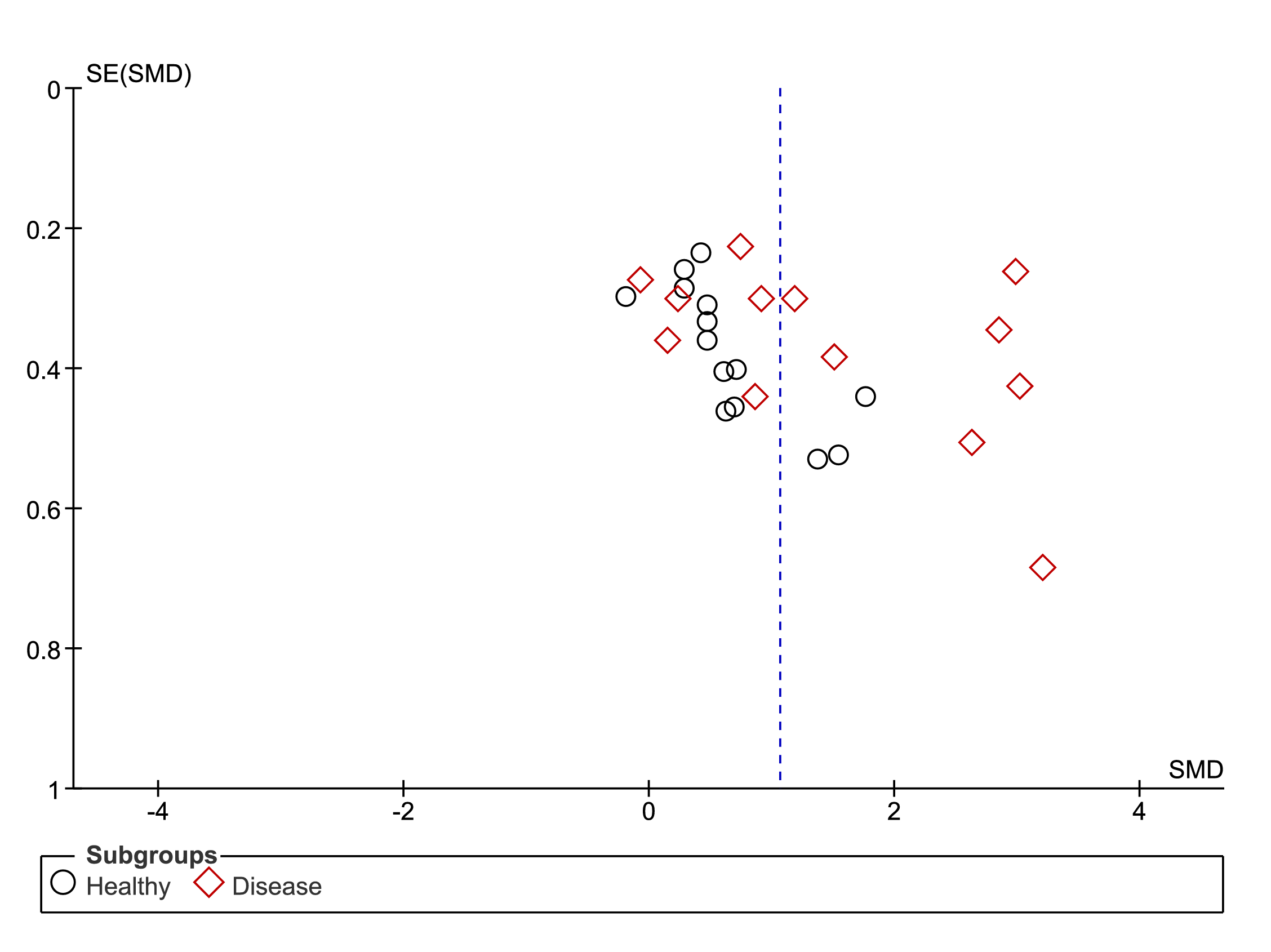


Supplementary Material 10E. Funnel plot of comparison: exercise versus control; outcome: serum klotho concentration in healthy and diseased subjects after chronic exercise.

**Regression-based Egger test for small-study effects**

Random-effects model

Method: DerSimonian–Laird

| Subgroup: Healthy  H0: beta1 = 0; no small-study effects  beta1 = 5.84  SE of beta1 = 3.123  t = 1.87  Prob > \|t\| = 0.0882 | Subgroup: Disease  H0: beta1 = 0; no small-study effects  beta1 = 4.08  SE of beta1 = 1.149  t = 3.55  Prob > \|t\| = 0.0040* |
| --- | --- |

* |t| ≤ 0.05 = statistically significant


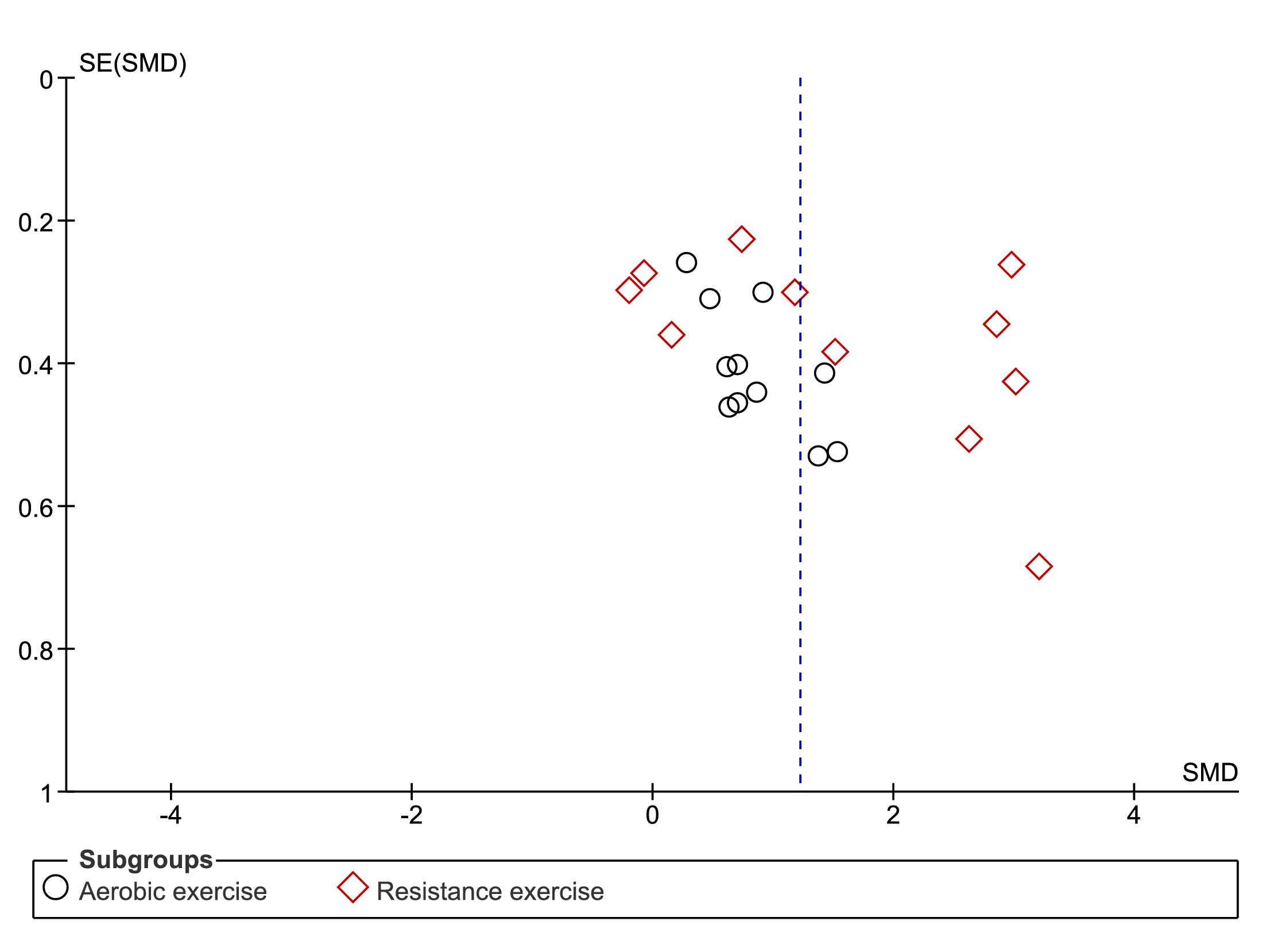


Supplementary Material 10F. Funnel plot of comparison: exercise versus control; outcome: serum klotho concentration after chronic aerobic exercise and resistance exercise.

**Regression-based Egger test for small-study effects**

Random-effects model

Method: DerSimonian–Laird

| Subgroup: Aerobic exercise  H0: beta1 = 0; no small-study effects  beta1 = 3.32  SE of beta1 = 1.414  t = 2.35  Prob > \|t\| = 0.0435* | Subgroup: Resistance exercise  H0: beta1 = 0; no small-study effects  beta1 = 6.49  SE of beta1 = 3.536  t = 1.84  Prob > \|t\| = 0.0995 |
| --- | --- |

* |t| ≤ 0.05 = statistically significant


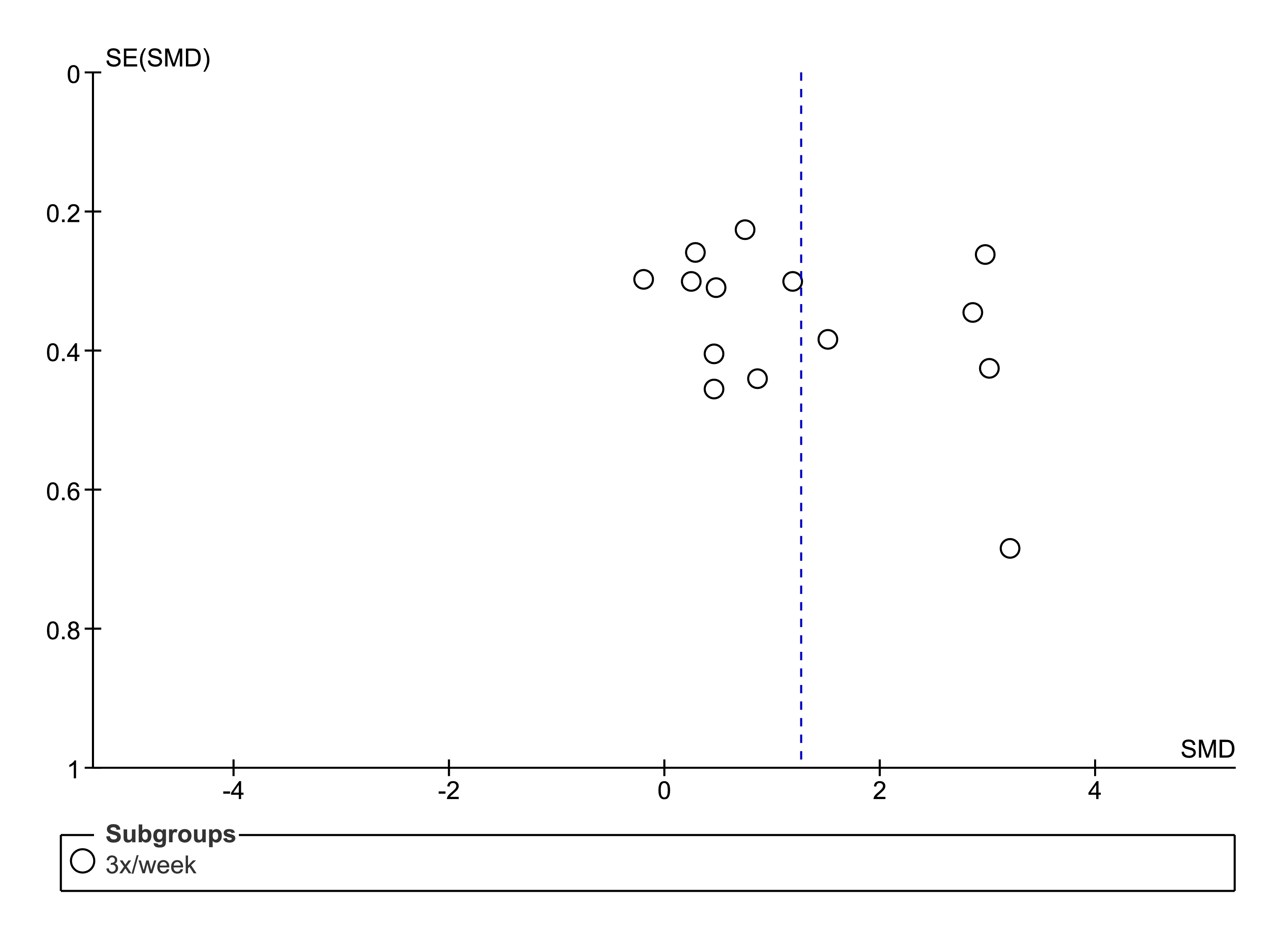


Supplementary Material 10G. Comparison funnel plot: exercise versus control; outcome: serum klotho concentration after chronic exercise three times per week.

**Regression-based Egger test for small-study effects**

Random-effects model

Method: DerSimonian–Laird

H0: beta1 = 0; no small-study effects

beta1 = 4.62

SE of beta1 = 3.185

t = 1.45

Prob > |t| = 0.1725
