# Supplementary material for: Effects of acute, subacute, and chronic exercise on plasma s-Klotho levels: a systematic review and meta-analysis
Source: J Physiol Biochem. 2026 May 2;82(1):46. doi: 10.1007/s13105-026-01182-2 (PMC13134988; doi:10.1007/s13105-026-01182-2)
Supplement: Supplementary file 11 — Supplementary file11 (DOCX 58 KB) [file 13105_2026_1182_MOESM11_ESM.docx]

|  |  | **Certainty assessment** | | | | | | |  | **Nº of patients** | |  | **Effect** |  |  |  |
| --- | --- | --- | --- | --- | --- | --- | --- | --- | --- | --- | --- | --- | --- | --- | --- | --- |
| Outcome | Comparison | Nº of studies | Study design | Risk of bias | Inconsistency | Indirectness | Imprecision | Publication bias |  | Exercise/pre | Control/post |  | Absolute (95% CI) |  | Certainty | Importance |
| s-Klotho^1^ | EX x CT | 27 | RCTs | serious^a^ | serious^b^ | not serious | not serious | detected |  | 699 | 534 |  | SMD 1.06 (0.69 - 1.43) |  | ⨁◯◯◯ Very low | Critical |
| s-Klotho^2^ | EX x CT | 8 | RCTs | serious^a^ | serious^b^ | not serious | not serious | N/A |  | 295 | 217 |  | SMD 1.87 (1.06 – 2.68) |  | ⨁⨁◯◯ Low | Critical |
| s-Klotho^3^ | EX (pre x post) | 2 | NRSs | serious^a^ | not serious | not serious | serious^c^ | N/A |  | 25 | 25 |  | SMD 0.04 (-0.52 - 0.59) |  | ⨁◯◯◯ Very low | Critical |
| s-Klotho^3^ | EX (pre x post) | 3 | RCTs | serious^a^ | not serious | not serious | serious^c^ | N/A |  | 32 | 32 |  | SMD 1.65 (0.84 - 2.47) |  | ⨁⨁◯◯ Low | Critical |
| s-Klotho^4^ | EX (pre x post) | 17 | NRSs | serious^a^ | not serious | not serious | serious^c^ | undetected |  | 321 | 321 |  | SMD 0.56 (0.31 - 0.81) |  | ⨁◯◯◯ Very low | Critical |
| s-Klotho^4^ | EX (pre x post) | 9 | RCTs/NRSs | serious^a^ | serious^b^ | not serious | serious^c^ | N/A |  | 143 | 143 |  | SMD 0.88 (0.32 - 1.44) |  | ⨁◯◯◯ Very low | Critical |
| s-Klotho^5^ | EX (pre x post) | 18 | RCTs/NRSs | serious^a^ | not serious | not serious | serious^c^ | detected |  | 338 | 338 |  | SMD 0.69 (0.41 - 0.97) |  | ⨁◯◯◯ Very low | Critical |
| s-Klotho^5^ | EX (pre x post) | 8 | RCTs/NRSs | serious^a^ | not serious | not serious | serious^c^ | N/A |  | 126 | 126 |  | SMD 0.62 (0.11 – 1.12) |  | ⨁◯◯◯ Very low | Critical |
| s-Klotho^6^ | EX (pre x post) | 21 | RCTs/NRSs | serious^a^ | not serious | not serious | serious^c^ | detected |  | 379 | 379 |  | SMD 0.60 (0.36 - 0.85) |  | ⨁◯◯◯ Very low | Critical |
| s-Klotho^6^ | EX (pre x post) | 4 | NRSs | serious^a^ | serious^b^ | not serious | serious^c^ | N/A |  | 73 | 73 |  | SMD 0.95 (-0.10 – 2.00) |  | ⨁◯◯◯ Very low | Critical |
| s-Klotho^6^ | EX (pre x post) | 1 | NRS | serious^a^ | N/A | not serious | very serious^d^ | N/A |  | 12 | 12 |  | SMD 1.08 (0.21 - 1.95) |  | ⨁◯◯◯ Very low | Critical |
| s-Klotho^7^ | EX x CT | 14 | RCTs | serious^a^ | not serious | not serious | not serious | undetected |  | 304 | 228 |  | SMD 0.57 (0.33 – 0.82) |  | ⨁⨁⨁◯ Moderate | Critical |
| s-Klotho^7^ | EX x CT | 13 | RCTs/NRSs | serious^a^ | serious^b^ | not serious | not serious | detected |  | 395 | 306 |  | SMD 1.51 (0.87 – 2.16) |  | ⨁◯◯◯ Very low | Critical |
| s-Klotho^8^ | EX x CT | 11 | RCTs/NRSs | serious^a^ | not serious | not serious | serious^c^ | detected |  | 216 | 148 |  | SMD 0.76 (0.52 - 1.00) |  | ⨁◯◯◯ Very low | Critical |
| s-Klotho^8^ | EX x CT | 11 | RCTs | serious^a^ | serious^b^ | not serious | not serious^c^ | undetected |  | 353 | 273 |  | SMD 1.60 (0.81 - 2.38) |  | ⨁⨁◯◯ Low | Critical |
| s-Klotho^8^ | EX x CT | 5 | RCTs | serious^a^ | not serious | not serious | serious^c^ | N/A |  | 130 | 113 |  | SMD 0.37 (0.12 – 0.63) |  | ⨁⨁◯◯ Low | Critical |
| s-Klotho^9^ | EX (pre x post) | 6 | RCT/NRSs | serious^a^ | not serious | not serious | serious^c^ | N/A |  | 64 | 64 |  | SMD 1.18 (0.42 – 1.94) |  | ⨁◯◯◯ Very low | Critical |
| s-Klotho^9^ | EX (pre x post) | 2 | NRSs | serious^a^ | not serious | not serious | serious^c^ | N/A |  | 60 | 60 |  | SMD 0.63 (-0.55 - 1.82) |  | ⨁◯◯◯ Very low | Critical |
| s-Klotho^9^ | EX (pre x post) | 1 | NRSs | serious^a^ | N/A | not serious | very serious^d^ | N/A |  | 19 | 19 |  | SMD -0.04 (-0.68 - 0.60) |  | ⨁◯◯◯ Very low | Critical |
| s-Klotho^10^ | EX x CT | 5 | RCTs | serious^a^ | not serious | not serious | serious^c^ | N/A |  | 87 | 69 |  | SMD 0.56 (0.23 - 0.89) |  | ⨁⨁◯◯ Low | Critical |
| s-Klotho^10^ | EX x CT | 14 | RCTs | serious^a^ | serious^b^ | not serious | not serious | undetected |  | 424 | 311 |  | SMD 1.26 (0.65 - 1.86) |  | ⨁⨁◯◯ Low | Critical |
| s-Klotho^10^ | EX x CT | 4 | RCTs | serious^a^ | serious^b^ | not serious | serious^c^ | N/A |  | 83 | 55 |  | SMD 1.18 (0.18 -2.18) |  | ⨁◯◯◯ Very low | Critical |
| s-Klotho^10^ | EX x CT | 1 | RCT | serious^a^ | N/A | not serious | very serious^d^ | N/A |  | 23 | 32 |  | SMD -0.08 (-0.61 - 0.46) |  | ⨁◯◯◯ Very low | Critical |

Supplementary Material 11. GRADE assessment summary.

^a^ Risk of bias ranged from moderate to high according to RoB 2

^b^ Lack of overlap of confidence intervals between studies in the forest plot

^c^ The sample size is below the optimal information size (n=400)

^d^ Sample size substantially below the optimal information size and based on a single study

^1^ Comparison: exercise versus control; outcome: s-klotho concentration after chronic exercise

^2^ Comparison: pre-intervention versus post-intervention; outcome: s-klotho concentration after chronic exercise on subjects with chronic kidney disease

^3^ Comparison: pre-intervention versus post-intervention; outcome: s-klotho concentration after acute /subacute exercise on subjects with type II diabetes

^4^ Comparison: pre-intervention versus post-intervention; outcome: s-klotho concentration after acute and subacute exercise

^5^ Comparison: pre-intervention versus post-intervention; outcome: s-klotho concentration in healthy and diseased subjects after acute and subacute exercise

^6^ Comparison: pre-intervention versus post-intervention; outcome: s-klotho concentration after acute and subacute aerobic exercise, resistance exercise or combination

^7^ Comparison: exercise versus control; outcome: s-klotho concentration in healthy and diseased subjects after chronic exercise

^8^ Comparison: exercise versus control; outcome: s-klotho concentration after chronic aerobic exercise, resistance exercise or combination

^9^ Comparison: pre-intervention versus post-intervention; outcome: s-klotho concentration after subacute exercise performed three, four and five times per week

^10^ Comparison: exercise versus control; outcome: s-klotho concentration after chronic exercise performed two, three, four and five times per week

Abbreviations: EX = exercise; CT = control; RCT = randomized controlled trial; NRS = non-randomized study; CI = confidence interval; SMD = standardized mean difference; N/A = not applicable.
